# Overcoming therapeutic challenges in acute myeloid leukemia: active targeting strategies by nano-drug delivery systems

**DOI:** 10.1186/s12967-026-07792-0

**Published:** 2026-02-07

**Authors:** Yuqian Tang, Jiaxin Li, Wu Ye, Yiwen Du, Ying Zhang, Yunxia Ye, Yuping Gong

**Affiliations:** 1 Department of Hematology, Hematology Research Laboratory, West China Hospital, Sichuan University, #37 GuoXue Xiang Street, Chengdu, 610041 China; 2 Key Laboratory of Drug-Targeting and Drug Delivery System of the Education Ministry and Sichuan Province, Sichuan Engineering Laboratory for Plant-Sourced Drug and Sichuan Research Center for Drug Precision Industrial Technology, West China School of Pharmacy, Sichuan University, Chengdu, 610041 China; 3 National-Local Joint Engineering Research Center of Biodiagnostics & Biotherapy, The Second Affiliated Hospital of Xi’an Jiaotong University, Xi’an, 710000 China

**Keywords:** Acute myeloid leukemia, Nano-drug delivery system, Nanomedicine, Target therapy

## Abstract

**Background:**

Acute myeloid leukemia (AML) is a highly aggressive hematological malignancy characterized by poor overall survival and high relapse rates. The standard chemotherapy remains the conventional “7+3” regimens, while the suboptimal pharmacokinetics and significant systemic toxicity in AML present ongoing challenges to long-term disease control. Nano-drug delivery systems (NDDSs) have emerged as a promising strategy to overcome these barriers by enabling enhanced drug stability, targeted delivery, and specific distribution. Although several NDDS-based therapies have been approved by FDA, the clinical translation of nanomedicine in AML remains limited. This is largely due to the unique pathophysiology of AML, which lacks the vascular structures found in solid tumors, resulting in a limited and atypical enhanced permeability and retention (EPR) effect. Active targeting strategies, including antibody, aptamer, and peptide-based ligand modifications, offer a compelling approach to improve cellular specificity and therapeutic efficacy.

**Methods:**

In this review, we provide a comprehensive overview of NDDSs engineered for AML, focusing on recent advances in active targeting approaches, their mechanistic advantages, and translational challenges.

**Results:**

Current active-targeting NDDSs in AML generally follow two major directions. One direction focuses on surface receptors that are aberrantly overexpressed on AML cells, thereby improving payload specificity. The other direction focuses on bone marrow (BM)-targeted nanocarriers that utilize cell homing mechanisms and disease-associated markers of the BM microenvironment.

**Conclusion:**

NDDSs designed for different targets, carrier materials, and release mechanisms have demonstrated improved pharmacodynamic effects, but they remain at the preclinical stage. Based on a summary of the current challenges facing NDDSs, this review further discusses key directions for next-generation system design, such as the development of personalized carriers, reduction of off-target effects, and more effective delivery to leukemia stem cells.

## Introduction

Acute myeloid leukemia (AML) is a highly aggressive hematologic malignancy characterized by clonal expansion of undifferentiated myeloid precursors, leading to the disruption of normal hematopoiesis. The disease progresses rapidly, with high relapse rates and poor overall survival, especially among elderly and unfit patients [1–3]. The standard first-line “7+3” regimen, consisting of 7 days of cytarabine (Ara-C) and 3 days of an anthracycline, has remained largely unchanged for decades [4, 5]. Despite the incorporation of molecularly targeted agents, including FLT3 inhibitors (midostaurin, gilteritinib, quizartinib) [6–8], IDH1/2 inhibitors (ivosidenib, enasidenib) [9, 10], and BCL-2 inhibitors (venetoclax) [11], long-term outcomes remain unfavorable. Hematopoietic stem cell transplantation (HSCT) offers curative potential but is limited by donor availability, toxicity, and cost [12, 13]. Immunotherapeutic approaches such as monoclonal antibodies (mAbs), antibody-drug conjugates (ADCs), and CAR-T cell therapy are emerging, yet they are associated with significant adverse events and variable efficacy in AML due to antigen heterogeneity and immune evasion [14, 15].

Key challenges in AML therapy are the systemic dissemination, as well as the pronounced genetic and phenotypic heterogeneity. Traditional chemotherapy lacks specificity and causes off-target toxicities, while small-molecule inhibitors are only effective in patients with select mutations and may face resistance. These limitations underscore the urgent need for more precise and personalized therapeutic strategies.

In this context, nano-drug delivery systems (NDDSs) have gained significant attention in oncology for their ability to improve pharmacokinetics, enhance tumor specificity, and minimize systemic toxicity [16–18]. NDDSs such as liposomes, polymeric micelles, dendrimers, and antibody-conjugated nanocarriers have demonstrated therapeutic potential in various tumors, including AML. For example, CPX-351, a liposomal co-formulation of Ara-C and daunorubicin (DNR), has been approved by the FDA for high-risk AML subtypes, demonstrating improved survival compared to conventional chemotherapy [19, 20].

In AML, passive targeting through the enhanced permeability and retention (EPR) effect can result in nanoparticle accumulation in the splenic and hepatic sinusoids, as well as inflamed bone marrow niches [21–23]. Nevertheless, this level of passive targeting is insufficient to ensure effective drug delivery, as leukemia cells (LCs) circulate freely in the bloodstream and infiltrate organs rather than forming solid tumor masses. Therefore, this fundamental difference necessitates the development of active targeting strategies. AML cells aberrantly overexpress specific surface receptors, allowing NDDSs to achieve precise drug delivery through receptor-ligand interactions. This approach overcomes the non-specific distribution of conventional chemotherapeutic agents. In parallel, by moving beyond a precision medicine paradigm that relies solely on intracellular genetic mutations, it also offers a new strategy to address the marked heterogeneity of AML. Consequently, this strategy has the potential to shift the current subtype-specific treatment model toward a more broadly applicable therapeutic platform with improved efficacy and reduced toxicity, ultimately increasing remission rates, lowering relapse risk, and improving long-term survival.

**Fig. 1:**
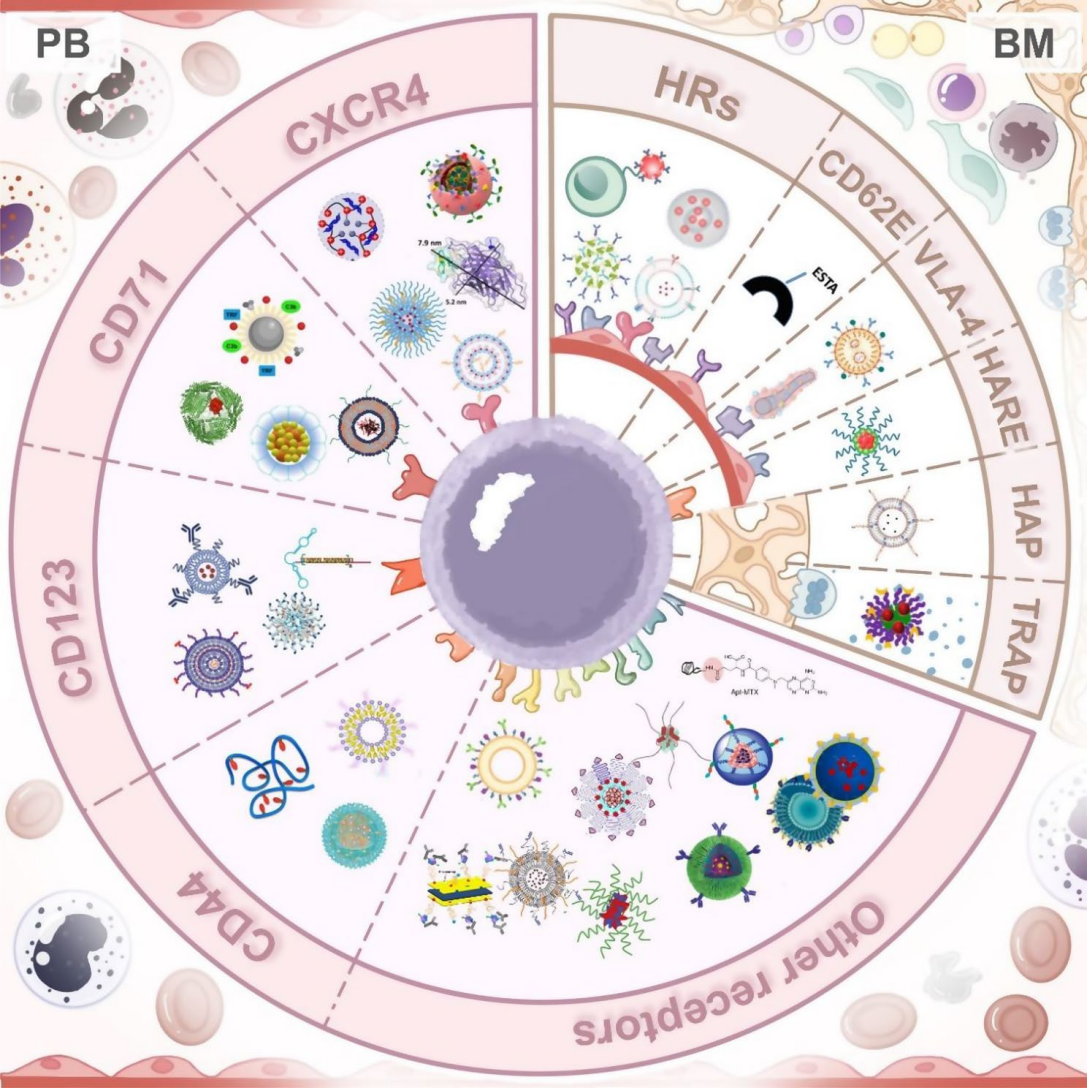
Schematic overview of targeted drug delivery strategies for the treatment of acute myeloid leukemia

In this review, we summarize the current landscape of active targeting strategies employed in NDDSs for AML treatment (Fig. 1), including available target receptor, nanocarrier engineering, and delivery mechanisms. We further discuss preclinical progress, translational obstacles, and potential solutions for promoting nanomedicine into AML clinical practice. Our aim is to provide a comprehensive framework for understanding and guiding the future development of precision-targeted nanomedicines in the treatment of AML.

## NDDSs for AML cells-specific delivery

Active targeting strategies utilizing AML surface receptors have emerged as a promising approach to achieve precise therapy. This section focuses on specific receptors that are aberrantly overexpressed on AML blasts but are low or absent on normal cells. The corresponding receptors and their nanocarrier applications are summarized in Table 1.

**Table 1:** Actively targeted drug delivery systems based on leukemic cell surface receptors

Target	Ligand	Cargo	Carrier	Stimuli	Animal model			Year	Ref
					Type	Cell	Site		
CXCR4	E5	E5	Micelle	-	CDX (SRM)	AE&CKIT^D816V^-transduced splenocytes	Disseminated	2020	[24]
CXCR4	E5	E5+DOX	Micelle	-	CDX (SRM)	AE&CKIT^D816V^-transduced splenocytes	Disseminated	2022	[25]
CXCR4	E5	Fe_3_O_4_ and Pt	Nanozyme	pH	CDX	HL-60	Disseminated	2021	[26]
CXCR4/CD44	E5/HA	DNR	PBNP	-	CDX	HL-60	Disseminated	2021	[27]
CXCR4	E5	DNR + Ara-C	HMPBs	Laser	-	-	-	2021	[28]
CXCR4	E5	E5+AraN	Liposome	-	CDX	HL-60-Luc	Disseminated	2024	[29]
CXCR4	T22	mRTA	NCs	-	CDX	THP-1-Luc	Disseminated	2018	[30]
CXCR4	T22	MMAE	NCs	-	CDX	THP-1-Luc	Disseminated	2020	[31]
CXCR4	T22	Ara-C	NCs	-	CDX	THP-1-Luc	Disseminated	2022	[32]
CXCR4	T22	DITOX	NCs	-	CDX	THP-1-Luc	Disseminated	2021	[33]
CXCR4	T22	PE24	NCs	-	CDX	MONO-MAC-6-Luc	Disseminated	2023	[34]
CXCR4	T22	MMAE	NCs	-	CDX	THP-1- Luc	Disseminated	2022	[35]
CXCR4/MUC1	T22-NLS/aptarmer	CRISPR-Case9 plasmid	Composite nanocarrier	-	-	-	-	2022	[36]
CXCR4	T22	VEN + SOR	Micelle	-	CDX	MV4-11-Luc	Disseminated	2024	[37]
CXCR4	AMD3100	AMD3100 + siRNA	PCX	-	GEM	*Cbfb-MYH11+ cells*	Disseminated	2020	[38]
CXCR4	CXCL12	Pt + DOX	Biomimetic NPs	-	CDX	HL-60	Disseminated	2022	[39]
CXCR4	CXCL12	DNR	Biomimetic NPs	-	-	-	-	2023	[40]
CXCR4	CXCL12/AMD3100	AMD3100+DOX+PDT	Biomimetic NPs	-	CDX	HL-60-Luc	Disseminated	2024	[41]
CD71	Tf	c-myb ASOs	Tf-polylysine	-	-	-	-	1992	[42]
CD71	Tf	GTI-2040	Tf-LPPs	pH	-	-	-	2010	[43]
CD71	Tf	*miR-29b*	Tf-LPPs	pH	CDX	MV4-11	Disseminated	2013	[44]
CD71	Tf	LOR-1248	Tf-NPs	-	CDX	MV4-11	Subcutaneous	2014	[45]
CD71	Tf	DOX	LPNs	-	-	-	-	2017	[46]
CD71	Tf	EDS	PEG-PLL-PLGA	-	-	-	-	2017	[47]
CD71/CD117	Tf/aptamer	DRN and Lut	Micelle	-	CDX	HL-60	Subcutaneous	2023	[48]
CD71/CD11b	Tf/C3b	DOX	FH	-	CDX	MOLM-13	Disseminated	2025	[49]
CD71	HumFt	Cytochrome C	HumFt	-	-	-	-	2019	[50]
CD71	Ferritin	*miRNA-145-5p*	HumFt-PAMAM	-	-	-	-	2021	[51]
CD71	Ferritin	ATO	Ferritin	-	CDX	Luc-HL-60	Disseminated	2021	[52]
CD71	HFn	Ara-C	HFn	-	CDX	Luc-HL-60	Disseminated	2023	[53]
CD71	HFn	siRNA	HFn	-	-	-	-	2024	[54]
CD71	OKT9	DOX	Liposome	-	-	-	-	1997	[55]
CD123	PO-6	PO-6	Micelle	-	CDX (SRM)	AE&CKIT^D816V^-transduced splenocytes	Disseminated	2021	[56, 57]
CD123	mAb	DNR	Liposome	-	-	-	-	2017	[58]
CD123	mAb	DNR	Niosome	-	CDX	THP-1	Disseminated	2017	[59]
CD123/CD33	mAb	DNR	Liposome	-	-	-	-	2019	[60]
CD123	Fab’ fragment	siRNA	Micelle	-	-	-	-	2017	[61]
CD123	ZW25	DNR	TDT	-	CDX	MOLM-13	Subcutaneous	2017	[62]
CD123	SS30	SS30	Hydrogel	Cas9/sgRNA	CDX	MOLM-13	Subcutaneous	2021	[63]
CD44	HA	Cur	Liposome	-	CDX	KG-1	Disseminated	2017	[64]
CD44	HA	6-MP	Prodrug	GSH	CDX	OCI-AML-2	Subcutaneous	2017	[65]
CD44	HA	DOX	LACHA	GSH	CDX	AML-2	Subcutaneous	2017	[66]
CD44	HA	HDC	GDH	-	-	-	-	2017	[67]
CD44	HA	DOX+GA	LPHNs	-	CDX	HL-60	Subcutaneous	2019	[68]
CD44	mAbs		PLGA NPs	-	-	-	-	2019	[69]
CD33	scFv	siRNA	Liposome	-	-	-	-	2010	[70]
CD33	mAbs	PMI	LONp	-	-	-	-	2018	[71]
CD33	Fab’ fragment	Ara-C	Liposome	pH	CDX	HL-60	Disseminated	2009	[72, 73]
CD33	scFv	GTI-2040	LNPs	-	CDX	Kasumi-1	Subcutaneous	2015	[74]
CD33	mAbs	G3139	aCD33-NKSN	-	CDX	Kasumi-1	Subcutaneous	2022	[75]
CD33	Aptamer	ASO	Gold NPs	-	-	-	-	2016	[76]
CD33	mAbs	ASO	RBCEVs	-	CDX	MOLM13-Luc-GFP	Disseminated	2022	[77]
CD33	mAbs	-	MoS_2_	-	-	-	-	2021	[78]
CD38	Dara	TRAIL	Liposome	-	CDX	OCI-AML2	Disseminated	2023	[79]
CD38	Dara	Vincristine	Polymersomal	-	CDX	MOLM-13-Luc	Disseminated	2023	[80]
FR	FA	DOX	Liposomes	-	CDX	KG-1	Ascites	2002/2007/2010	[81–83]
FR	FA	SiRNA	Albumin	-	CDX	KG-1	Subcutaneous	2021	[84]
FR	FA	DNR + Eme	Liposomes	-	-	-	-	2014	[85]
FR	FA	PTX TanIIA	LB-MSN	-	CDX	NB4	Subcutaneous	2020	[86]
FLT3	FLT3L	*miR-150*	Dendrimers	-	GEM	MLL-AF9-transduced BM	Disseminated	2016	[87]
FLT3	scFv	scFv	ELP	-	CDX	MOLM-13	Disseminated	2020	[88]
FLT3/CD99	scFv	scFv	ELP	-	CDX	MV4-11	Disseminated	2024	[89]
CD117	mAb	DM1	ADC	-	-	-	-	2018	[90]
CD117	mAb	DM1	ADC	-	CDX	Kasumi-1	Subcutaneous	2022	[91]
CD117	Aptamer	MTX	Aptamer-drug conjugate	-	-	-	-	2015	[92]
CLL-1	CLL1-L1	DNR	Micelle	-	-	-	-	2012	[93]
CLL-1	CLL1-L1	DNR	Micelle	-	PDX	Primary cells	Disseminated	2019	[94]
CD34	mAb	DOX	Liposome	-	-	-	-	2004	[95]
CD99	scFv	scFv	ELP	-	CDX	MOLM-13	Disseminated	2020	[96]
CD96	mAbs	ICG	CPSNPs	-	CDX	32D-p210-GFP	Disseminated	2011	[97]

Abbreviations: CDX, cell-derived xenograft; SRM, secondary recipient model; GEM, genetically engineered mouse. HFn, heavy ferritin chain; ICG, indocyanine green. Others are defined in the Abbreviations section

Among the best-characterized targets, CXCR4, CD123, CD44, and CD71 have garnered significant attention due to their roles in leukemic cell survival, proliferation. CXCR4, CD123, and CD44 are involved in intracellular signaling pathways critical to AML pathogenesis. Notably, drug delivery systems directed at these receptors not only facilitate targeted payload delivery but may also confer direct antileukemic effects via receptor blockade. In contrast, CD71 (transferrin receptor 1) is primarily associated with cellular iron metabolism and proliferation. Its high expression in AML, particularly among aggressive and proliferative subtypes, renders it an attractive target for therapeutic intervention.

Various targeting ligands, including monoclonal mAbs, peptides, aptamers, and polysaccharide derivatives, have been designed to engage these receptors, with each type offering distinct advantages in terms of affinity, stability, and translational feasibility. For example, antibodies exhibit high affinity but are limited by high immunogenicity, large molecular size, and high cost. Nucleic acid aptamers are easy to synthesize but are prone to degradation in vivo. In comparison, peptides combine high affinity with ease of synthesis, while also offering low immunogenicity, controlled chemical synthesis, good batch-to-batch consistency, and facile modification. However, peptides can be vulnerable to proteolytic cleavage. The selection of an optimal ligand thus depends on a comprehensive consideration of the intended application, required pharmacokinetics, and translational practicality.

### C-X-C chemokine receptor 4 (CXCR4)

CXCR4 is a G-protein coupled receptor with seven transmembrane domains that binds stromal derived factor 1α (SDF-1α or CXCL12) [98]. This receptor-ligand interaction plays a crucial role in directing LCs homing and retention in BM and spleen niches, where LCs receive survival and anti-apoptotic signals from stromal support [99, 100]. Overexpression of CXCR4 is frequently observed in AML and is associated with poor prognosis and chemotherapy resistance [101, 102]. Consequently, the CXCR4/CXCL12 axis has become an attractive therapeutic target for active-targeting nanomedicine strategies.

**Fig. 2:**
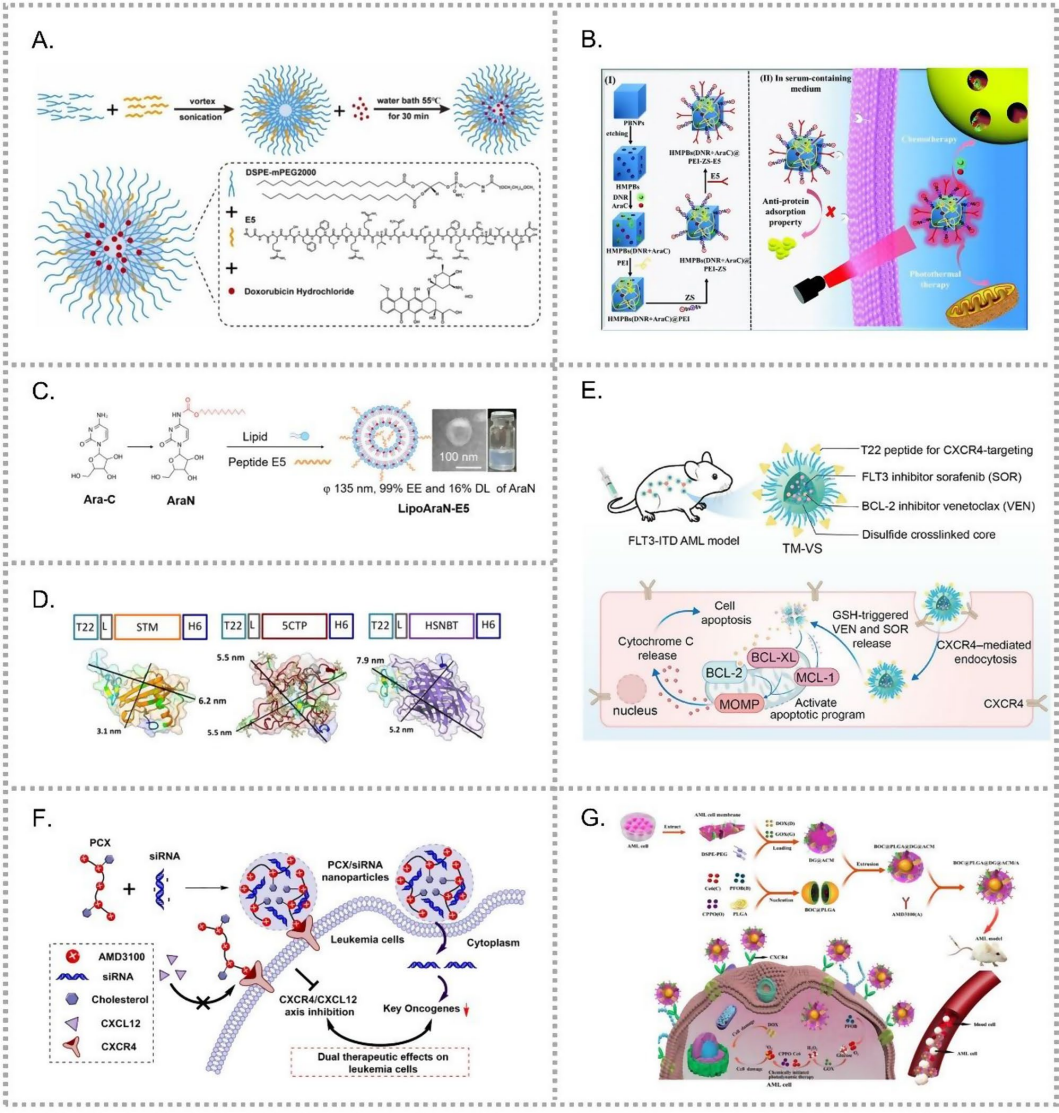
CXCR4-targeted nano-drug delivery systems (NDDSs) for AML therapy. (**A**) E5-modified micelles co-loaded with doxorubicin (M-E5-DOX), enabling CXCR4-targeted delivery, inhibition of the CXCR4/CXCL12 axis, and direct elimination of leukemic cells [25]. (**B**) CXCR4-targeted, photothermal-responsive HMPB co-loaded with DNR and Ara-C, surface-modified with zwitterionic sulfobetaine (ZS) to reduce nonspecific interactions, and conjugated with E5 peptide for leukemia cell targeting [28]. (**C**) E5-modified liposomes (LipoAran) co-loaded with a hydrophobic Ara-C derivative, enabling CXCR4-targeted delivery and enhanced liposomal stability. (Adapted with permission from [29]. Copyright 2024 American chemical society.) (**D**) T22-based protein nanoconjugates incorporating human-derived scaffolds for CXCR4-targeted delivery of cytotoxic agents with enhanced in vivo stability and reduced immunogenicity [35]. (**E**) T22-decorated, disulfide-crosslinked polymeric micelles for CXCR4-targeted co-delivery of venetoclax and sorafenib with enhanced synergistic efficacy. (Adapted with permission from [37]. Copyright 2024 American chemical society.) (**F**) Cholesterol-modified polymeric CXCR4 antagonist (PCX) enables CXCR4 blockade and siRNA delivery with nuclease protection [38]. (**G**) Biomimetic nanoplatform (BOC@PLGA@DG@ACM/A) for AML-targeted co-delivery of DOX, GOX, and PDT agents with enhanced therapeutic efficacy. (Adapted with permission from [41]. Copyright 2024 American chemical society.)

One of the most widely studied approaches involves the CXCR4-antagonistic peptide E5. Meng et al. synthesized E5 and fabricated micelles (M-E5) with DSPE-PEG [24]. M-E5 bound efficiently to CXCR4 at the ECL1/ECL2 and N-terminal domains, which overlap with CXCL12 binding sites. By downregulating proliferation and adhesion genes and upregulating apoptosis and differentiation genes, M-E5 blocked the CXCR4/CXCL12 axis and mobilized LCs into peripheral blood (PB). Building on this, a co-delivery micelle (M-E5-DOX) was created by loading doxorubicin (DOX) with E5 using DSPE-mPEG2000 (Fig. 2A) [25]. This co-delivery system integrates three key functions: CXCR4 targeting, CXCR4/CXCL12 axis inhibition and direct LCs elimination.

Zhang’s group further advanced this strategy by combining E5 with nanozymes to form Fe3O4@Pt@E5 [26]. Fe_3_O_4_ and Platinum (Pt) catalyzed H_2_O_2_ into ·OH under acidic pH lysosomal conditions, inducing LCs death. They also designed a CXCR4/CD44 dual-target Prussian blue nanoparticles (PBNPs) to deliver DNR [27]. Further, a multifunctional system, HMPBs (DNR + AraC)@PEI-ZS-E5, was developed. It incorporated hollow mesoporous Prussian blue (HMPB) NPs with zwitterionic modification for stability and E5 for targeting (Fig. 2B) [28]. Despite its promising design, this system was only validated in vitro. Challenges for in vivo translation include blood dilution effects, optimization of laser power for photothermal therapy, and evaluation of whether localized bone marrow exposure can achieve sufficient therapeutic efficacy in the context of disseminated leukemia cells.

Other formulations have explored E5 as both targeting ligand and CXCR4 blocker. For example, LipoAraN-E5 was developed as a dual-function liposomal formulation that co-loads E5 and AraN, an amphiphilic Ara-C derivative generated by C14 conjugation (Fig. 2C). This design resulted in a structurally stable liposomal system [29].

Another notable ligand is T22, derived from polyphemusin II of the horseshoe crab. T22 functions both as a CXCR4 antagonist and a structural element in self-assembling nanoparticles. Compared to the existing protein drug delivery system, mostly ADCs, T22-based NPs offer superior in vivo stability, lower renal filtration, and reduced proteolysis. A Spanish group engineered T22-tagged nanoconjugates (NCs) such as T22-mRTA-H6 [30], T22-GFP-H6-MMAE [31] and T22-GFP-H6-Ara-C [32], achieving potent antitumor effects with low systemic toxicity in CXCR4-overexpressing AML cells. Further systems, including T22-DITOX-H6 [33] and T22-PE24-H6 [34], incorporated bacterial toxins but faced immunogenicity issues. To overcome this, human protein scaffolds such as Stefin A, chorionic gonadotropin, and Nidogen G2 were employed, generating NPs like T22-STM-H6-MMAE, which retained efficacy with reduced off-target effects (Fig. 2D) [35].

Beyond peptide-based designs, Ren et al. developed a dual-target nanomedicine (P@PPM) combining CXCR4 and MUC1 aptamers with CRISPR-associated protein 9 (Cas9) cargo to downregulate CXCR4 expression and induce LC death [36]. T22 was also recently incorporated into a disulfide cross-linked polymeric micelle designed to co-deliver VEN and sorafenib (SOR) (Fig. 2E) [37], achieving enhanced therapeutic effects at reduced drugs dosages by optimizing the drug ratio.

Small-molecule CXCR4 antagonists have also been repurposed. AMD3100 (Plerixafor), modified with cholesterol, yielded polymeric CXCR4 antagonist (PCX) (Fig. 2F) [38]. PCX both inhibited CXCR4 to sensitize LCs and served as a carrier for small interference RNA (siRNA), protecting it from nuclease degradation.

In addition to these synthetic systems, biomimetic strategies have also been developed for CXCR4-targeted delivery. Kong et al. constructed cell membrane-derived nanoparticles encapsulating PFOB@PLGA@Pt and DOX (PFOB@PLGA@Pt@DOX-CM) [39]. These particles converted H_2_O_2_ into reactive oxygen species (ROS), killed LCs, inhibited BM infiltration, and homed to BM niches. Another design, HMPB(DNR)@CM NPs, combined DNR-loaded Prussian blue cores with BMSC membranes, achieving CXCR4 targeting and ROS scavenging to protect the liver from DNR [40]. Recently, a multi-mechanism platform, BOC@PLGA@DG@ACM/A, integrated photodynamic therapy (PDT), chemotherapy, and biomimetic targeting (Fig. 2G). It employed AML cell membranes decorated with AMD3100, DOX, and glucose oxidase, alongside a PLGA core encapsulating CPPO, PFOB, and the photosensitizer Ce6 [41].

In summary, CXCR4-targeted NDDSs span a diverse range of delivery platforms, from polymeric micelles and protein-based self-assembling systems to gene-editing nanocarriers and biomimetic constructs. These formulations collectively offer promising strategies for disrupting LC-niche interactions and enhancing therapeutic precision in AML.

### CD71

CD71, or transferrin receptor 1, is a 97-kDa type-II transmembrane glycoprotein that binds transferrin (Tf) and ferritin to mediate cellular iron uptake [103, 104]. Iron serves as an essential mental cofactor to regulate important cell process, such as cellular respiration, DNA synthesis and proliferation [105, 106]. Tumor cells, including AML cells, exhibit a heightened requirement for iron to support DNA synthesis during active proliferation [105–107]. Notably, CD71 expression in AML cells surpasses that observed in other types of leukemia [108–111], making it an attractive target for drug delivery. Moreover, anti-CD71 therapies have demonstrated anti-leukemia efficacy [112, 113], further supporting the feasibility of CD71 as a therapeutic target.

**Fig. 3:**
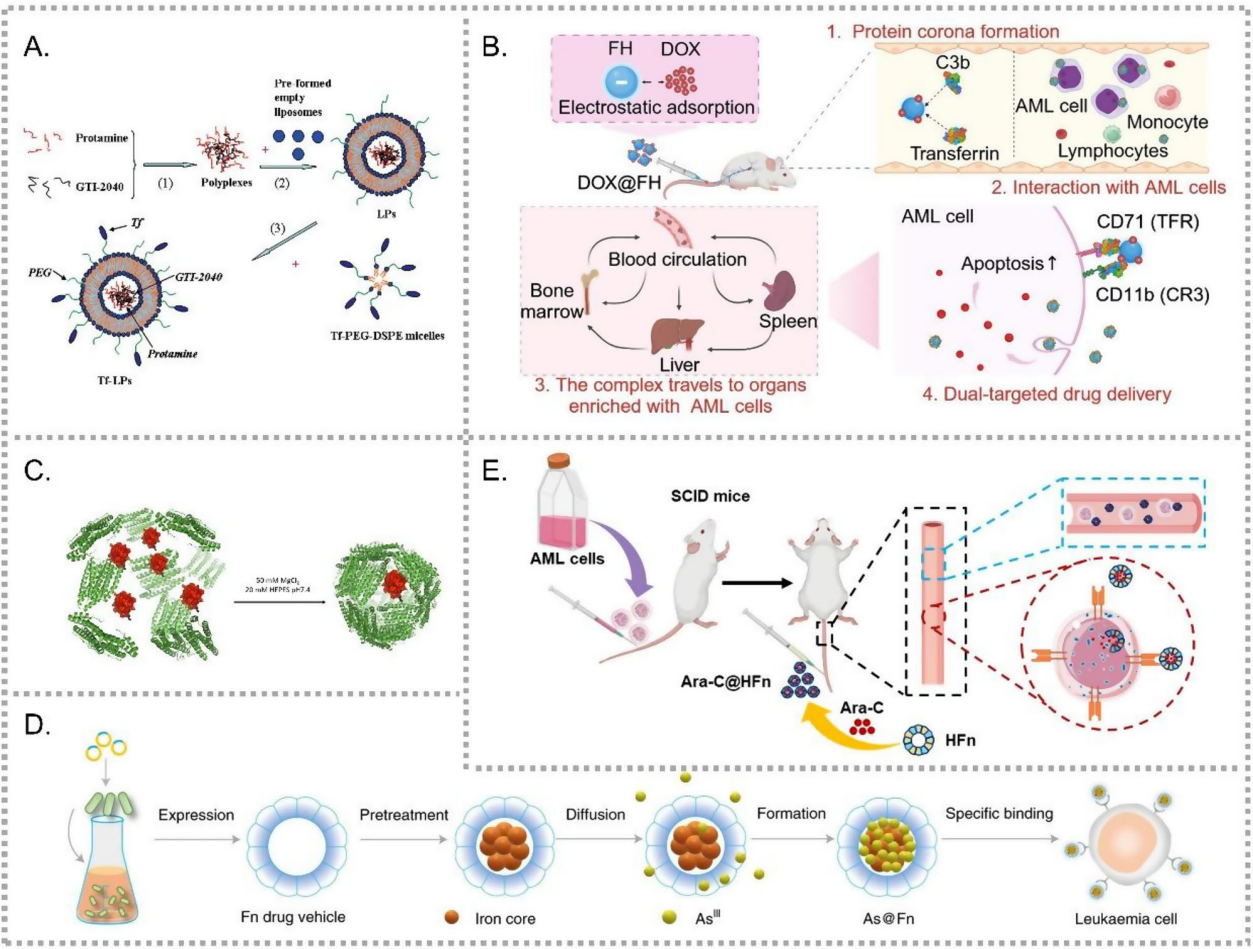
Nanocarriers for CD71-targeted drug delivery. (**A**) Tf-modified pH-sensitive lipopolyplex nanoparticles (LPPs) for targeted delivery of GTI-2040, an ASO targeting R2 subunit of RNR. (Adapted with permission from [43]. Copyright 2010 American chemical society.) (**B**) Feraheme (FH)-based nanoparticles with a protein corona of transferrin and C3b for dual targeting of CD71 and CD11b, enhancing DOX delivery and reducing AML burden in vitro and in vivo. (Adapted with permission from [49]. Copyright 2025 American chemical society.) (**C**) AfFt, a humanized *archaeoglobus* ferritin, enables CD71 targeting and efficient encapsulation of positively charged cargos, including cytochrome C [50]. (**D**) As@Fn nanoparticles, thermotolerant apoferritin loaded with ATO and iron, retain ATO in its trivalent form while exhibiting high CD71 affinity, highlighting their potential for large-scale production and clinical application [52]. (**E**) ferritin nanocages loaded with ara-C, demonstrating stable CD71 expression and effective delivery for leukemia treatment [53]

Tf is a key ligand for targeting CD71-high cells. In 1992, researchers used transferrin-polylysine to deliver c-*myb* antisense oligodeoxynucleotides (ASOs) [42]. Marcucci’s team further developed a series of Tf-based NPs for non-coding RNAs delivery. They first used Tf-modified pH-sensitive lipopolyplex nanoparticles (LPPs) to deliver GTI-2040, an ASO targeting the R2 subunit of ribonucleotide reductase (RNR), which later entered Phase I and II clinical trials (Fig. 3A) [43]. They later delivered *miR-29b* using transferrin-conjugated anionic LPPs [44], and utilized Tf-modified lipid nanoparticles (LNPs) to carry LOR-1248, a siRNA targeting RNR which showed strong antitumor activity [45].

Other Tf-modified systems also demonstrated efficacy. Zhu et al. synthesized a novel target ligand, transferrin-polyethylene glycol-oleic acid (Tf-PEG-OA), and prepared Tf-DOX P85/LPNs which effectively killed LCs while overcoming multidrug resistance [46]. Sun et al. developed Tf-modified PEG-PLL-PLGA micelles for edelfosine (EDS) delivery, achieving significant anti-leukemia effect [47]. Zhu et al. designed a binary nanodrug, combining Tf-Lut (Tf-modified, luteolin-loaded NPs) with AP-Drn (aptamer-decorated, DNR-loaded NPs), targeting CD71 and CD117 respectively [48]. Wu et al. exploited protein corona formation on feraheme (FH) to achieve dual targeting, as the corona contained both Tf and C3b. Using FH as a DOX carrier (DOX@FH), they achieved dual targeting of CD71 and CD11b and reduced AML burden in vitro and in vivo (Fig. 3B) [49].

Ferritins are emerging as promising nanocarriers for drug delivery due to their hollow structure and targeting ability to CD71. Bonamore’s team engineered humanized *Archaeglobus fulgidus* (AfFt) by grafting a loop from human H-ferritin onto AfFt (humanized *Archaeoglobus* ferritin, HumFt), enabling efficient CD71-targeting capability. HumFt retained the ability to efficiently encapsulate positively charged cargos, such as full-length cytochrome C (Fig. 3C) [50]. To enable the loading of negatively charged nucleic acids, polyamine dendrimers were incorporated into the HumFt cavity, enabling the delivery of *miRNA-145-5p* [51]. Considering the hollow cage and the natural absorptivity to Fe ions of apoferritin (ferritin without an iron core), Wang et al. utilized thermotolerant apoferritin to load arsenic trioxide (ATO) and iron, forming Fe-O-As cores inside the nanocages (Fig. 3D) [52]. The resultant As@Fn nanoparticles retained ATO in its medicinal trivalent form while exhibiting high CD71 affinity. This streamlined and safe preparation procedure highlighted the potential for large-scale production and clinical application. This team also leveraged ferritin nanocages to load Ara-C, demonstrating stable CD71 expression levels during treatment (Fig. 3E) [53].

Anti-transferrin receptor (TfR) mAbs offer another approach for CD71 targeting. Suzuki’s group developed OKT9-modified chemoimmunoliposomes (OKT9-CIL) encapsulating DOX [55], which specifically bound to CD71-positive LCs and maintained high DOX levels in K562/ADM cells, which are resistant to conventional DOX treatment.

Overall, diverse CD71-targeted delivery strategies have demonstrated effective drug delivery and antitumor activity in leukemia models. Among them, ferritins stand out for their dual role as carriers and targeting ligands, positioning CD71-targeted systems as a promising platform for AML therapy.

### CD123

CD123, also known as the interleukin-3 receptor alpha subunit (IL-3Rα), is a type-I cytokine receptor coded in the pseudo-autosomal region of Xp22.3 and Yp22.3 [114–116]. Upon binding IL-3, CD123 heterodimerizes with the common β-subunit of the GM-CSF/IL-5/IL-3 receptor complex, activating JAK/STAT and PI3K/mTOR pathways and upregulating anti-apoptotic proteins, thereby promoting cell differentiation and proliferation [116, 117]. CD123 is expressed in 40%–93% of leukemia cell samples, with variations likely due to detection methods or patient cohorts [118–121]. It is aberrantly overexpressed by CD34^+^CD38^-^ AML cells but undetectable or low in their normal BM counterparts [118, 119], enabling proliferation even under low IL-3 conditions [122]. Notably, only the CD34^+^/CD38^-^/CD123^+^ subpopulation is capable of initiating leukemia in immunodeficient mice, establishing CD123 as a marker of leukemia stem cells (LSCs) [123]. Moreover, CD123 expression is closely associated with poor prognosis in AML [124–128]. Given these features, CD123 is a compelling therapeutic target in AML.

**Fig. 4:**
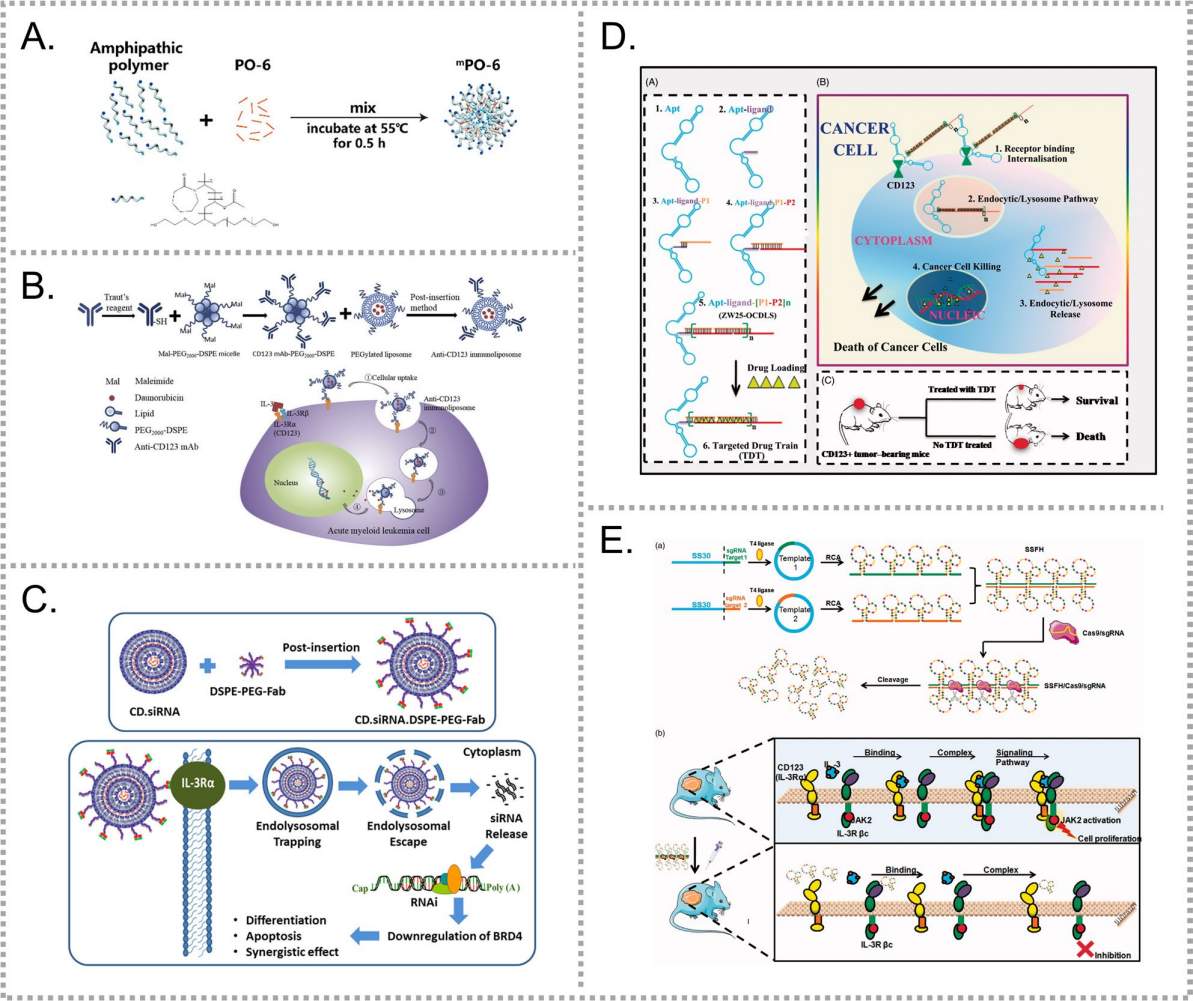
CD123-targeted nanomedicines for enhanced drug delivery and therapy. (**A**) The amphiphilic polymeric micelle (^m^PO-6) encapsulating the CD123-selective peptide PO-6 enhances cellular uptake and CD123/IL-3 axis blockade. (Adapted with permission from [57]. Copyright 2022 American chemical society.) (**B**) CD123-LIP, a CD123 antibody-conjugated DNR-loaded immunoliposome [58]. (**C**) CD.DSPE-PEG-Fab, a CD123 Fab-conjugated cyclodextrin-based NP for siRNA delivery and BRD4 silencing. (Adapted with permission from [61]. Copyright 2017 American chemical society.) (**D**) The CD123 aptamer-conjugated TDT enables selective targeting and high-efficiency DOX loading [62]. (**E**) SS30 polyaptamer hydrogel (SSFH) system releases SS30 via CRISPR/Cas9, enhancing therapeutic efficacy in AML cells [63]

Xu et al. successfully identified a CD123-selective binding peptide (PO-6) [56, 57], which competes with IL-3 for CD123 binding. When encapsulated in amphiphilic polymeric micelles (^m^PO-6, Fig. 4A), PO-6 exhibited improved solubility and cellular uptake. Beyond targeting, ^m^PO-6 mimicked a CD123 antibody, effectively blocking the CD123/IL-3 axis, resulting a significant anti-leukemia effect and prolonged survival time in mice. Besides blocking the CD123/IL-3 axis, PO-6’s targeting capability may also serve as a ligand for future drug delivery systems.

As an alternative strategy, Wang et al. developed antibody-based NDDSs. They conjugated a CD123 antibody onto a DNR-loaded liposome via a PEGylated linker, generating CD123-LIP (Fig. 4B) [58], and similarly designed an anti-CD123 niosome (CD123-NS) for DNR delivery [59]. To overcome limitations of single-targeting, such as relapse from target-negative cells, they further constructed a CD123/CD33 dual-antibody-modified liposome to broaden the targeting spectrum [60]. In parallel, Gou’s team constructed another CD123 antibody-decorated NDDS by conjugating the Fab fragment onto cyclodextrins through a PEGylated linker (CD.DSPE-PEG-Fab, Fig. 4C) [61]. This system enabled efficient siRNA delivery, overcoming challenges such as rapid clearance and poor intracellular trafficking, thereby achieving effective *BRD4* silencing and notable antitumor activity in vitro.

Given the sensitivity of antibodies to temperature, pH, and freeze-thaw cycles, Wu et al. applied systematic evolution of ligand exponential enrichment (SELEX) to generate high-affinity CD123 aptamers ZW25 and CY30 [62]. ZW25 was then incorporated into an aptamer-mediated targeted drug train (TDT) containing two C/G-rich probes and a pair of aptamer-linked trigger probes (Fig. 4D). This system exhibited high loading efficiency, as DOX efficiently intercalates into the C/G-rich regions at high concentration. The aptamers demonstrated selective binding to CD123, and the TDT based on them showed promising therapeutic efficiency. Furthermore, this team developed the first CD123 thioaptamer, SS30, exhibited high affinity for CD123 and compete with IL-3 at the CD123 binding cite, blocking multiple signaling pathways and inhibiting AML cells growth [129, 130]. To overcome SS30 instability in vivo, they designed a DNA hydrogel delivery platform, termed SS30 polyaptamer hydrogel (SSFH) (Fig. 4E). This hydrogel system enabled CRISPR-Cas9-mediated controlled release and enhanced therapeutic efficacy [63].

### CD44

CD44 is a non-kinase transmembrane glycoprotein encoded on chromosome 11 [131, 132], and it plays a critical role in normal myelopoiesis [133]. On progenitor cells, CD44 mediates adhesion to hyaluronic acid (HA), a glycosaminoglycan component of the extracellular matrix [134–136]. Blocking CD44 with mAbs has been shown to completely inhibit long-term myelopoiesis in bone marrow (BM) culture in vitro [135, 137]. Notably, CD44 is significantly overexpressed in various malignancies, including AML [138, 139]. Upon HA binding, CD44 becomes activated and recruits cytoplasmic adaptor proteins, initiating signaling pathways that support tumor cell survival [138]. Inhibition of this signaling consistently exerts anti-AML effects [140–142]. Together, these findings highlight CD44 as both a promising therapeutic target and a potential mediator for active drug delivery.

**Fig. 5:**
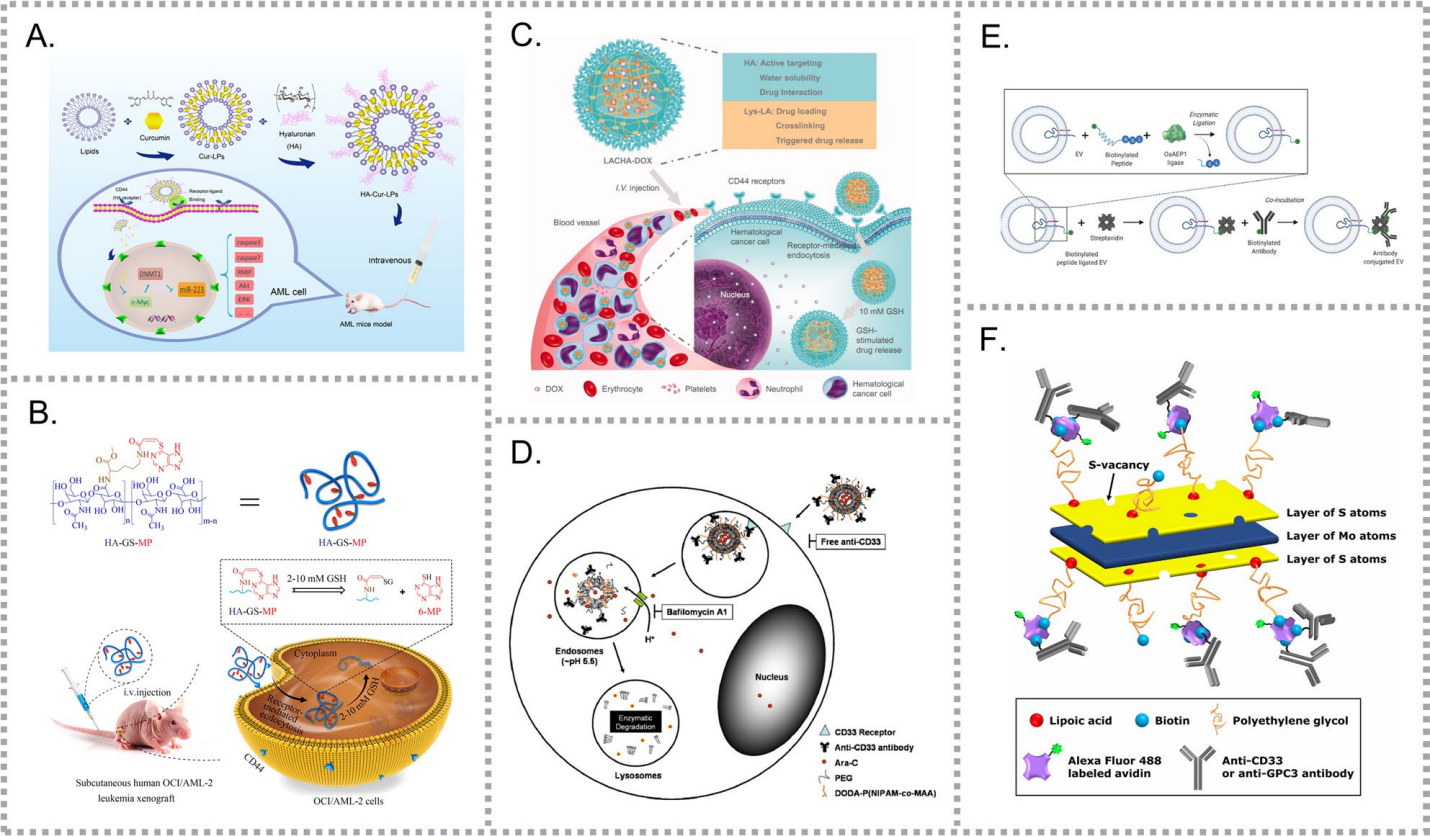
CD44- and CD33-targeted nanomedicines for AML therapy. (**A**) HA-Cur-LPs enable CD44-targeted curcumin delivery with therapeutic potential in AML. (Adapted with permission from [64]. Copyright 2017 American chemical society.) (**B**) HA-GS-MP enables GSH-responsive 6-MP release with improved solubility and AML inhibition. (Adapted with permission from [65]. Copyright 2017 American chemical society.) (**C**) LACHA-DOX delivers DOX via CD44 targeting, showing strong anti-AML effects in vitro and in vivo [66]. (**D**) Anti-CD33 fab-conjugated pH-sensitive liposomes enhance Ara-C delivery and retention in AML cells [72]. (**E**) CD33 antibody-conjugated RBCEVs enable targeted delivery of ASOs against FLT3-ITD and *miR-125b* while preserving vesicle integrity [77]. (**F**) Anti-CD33 MoS₂ nanoflakes for potential AML diagnosis and therapy [78]

HA, the natural ligand of CD44, has been widely applied in AML drug delivery. Sun et al. developed HA-modified liposomes for curcumin delivery (HA-Cur-LPs, Fig. 5A) [64], which exhibited high affinity and significant therapeutic potential. Qiu et al. designed HA-mercaptopurine prodrug (HA-GS-MP) to specifically target CD44-positive LCs [65]. HA-GS-MP was composed of HA conjugated to 6-Mercaptopurine (6-MP) via a glutathione (GSH)-responsive carbonyl vinyl sulfide linker, which enhanced the stability and water solubility of 6-MP while enabling rapid release and effective tumor inhibition (Fig. 5B). Zhong et al. developed another CD44-targeted nanocarrier by encapsulating DOX in lipoic acid-crosslinked HA (LACHA-DOX) (Fig. 5C), which demonstrated potent anti-AML effect both in vitro and in vivo [66]. Cherukula et al. developed a graphene quantum dots (GDH)-based system for histamine dihydrochloride (HDC) delivery to suppress ROS in LCs [67], where HA was conjugated to 3,4-dihydroxy-L-phenylalanine (DA) for stability, and then anchored onto HDC loaded GDH. Shao’s group designed lipid-polymer hybrid NPs for co-delivery of DOX and gallic acid (GA), using DSPE-PEG-HA to enable CD44 targeting [68].

In addition to HA-based approaches, CD44 mAbs have been employed to enhance target drug delivery. Noureldien’s team conjugated anti-CD44 mAbs to PLGA NPs encapsulating parthenolide (PTL) [69]. Their study showed that elevated NF-κB activity correlates with poor AML prognosis and that PTL, a natural NF-κB inhibitor, can be effectively delivered using this targeted approach.

### CD33

CD33, or Siglecs-3, is a 67-kDa transmembrane glycoprotein selectively expressed on myeloid linage, and its activation inhibits tyrosine phosphorylation and calcium mobilization [143–145]. In AML, CD33 is overexpressed in 85–90% of cases [146–149], with a molecule density in BM cells approximately 3.5-fold higher than in normal counterparts [150]. High CD33 expression is associated with poor prognosis [151, 152]. Therapeutic strategies targeting CD33, including mAbs and CD33-specific immunotoxin, have shown clinical potential in AML [153–155]. Notably, CD33-targeting therapies restore normal clonal hematopoiesis without impairing differentiation of CD33^–^CD34^+^ myeloid progenitors [154], supporting its value as a selective drug delivery target.

The most common strategy for targeting CD33-positive leukemic cells is antibody-based drug delivery. Rothdiener et al. developed siRNA-loaded liposomes targeting the AML1/MTG8 fusion protein, with an anti-CD33 single-chain variable fragment (scFv) conjugated to the liposomal surface to enhance specificity [70]. Anti-CD33 mAbs have also been applied to construct multifunctional carriers [71]. For instance, a humanized anti-CD33 mAbs was conjugated to fluorescent lanthanide oxyfluoride nanoparticles (LONps) co-loaded with a dual MDM2/MDMX peptide inhibitor (PMI), allowing disease tracking and treatment monitoring.

Given that the efficacy of Ara-C depends on intracellular bioavailability and sustained exposure, rapid clearance of free Ara-C increases the risk of drug resistance. To address this, anti-CD33 Fab fragments were conjugated to pH-sensitive liposomes for targeted Ara-C delivery (Fig. 5D) [72, 73]. Another approach modified LNP carrying GTI-2040, an ASO targeting mRNA of R2 subunit of RNR, with anti-CD33 scFv to overcome Ara-C resistance [74].

Additional CD33-targeted gene delivery strategies have also been explored. A vector (aCD33-NKSN) was designed to deliver the *BCL2* ASOs (G3139), incorporating a nuclear localization signal, fusion peptide, and stearic acid to enhance nuclear transport [75]. Considering the therapeutic potential of ASOs in AML, Zaimy et al. developed gold nanoparticles functionalized with five ASOs targeting key AML oncogenes, integrated with an anti-CD33^+^/CD34^-^ aptamer for precise targeting [76]. Furthermore, red blood cell-derived extracellular vesicles (RBCEVs) were engineered to deliver ASOs against FLT3-ITD and *miR-125b* [77]. In this system, CD33 antibodies were attached to RBCEVs through a biotin-streptavidin interaction while preserving vesicle membrane integrity (Fig. 5E).

Beyond drug and gene delivery, CD33-targeting strategies have also been applied in diagnostics. For example, an anti-CD33 antibody was conjugated to MoS_2_ nanoflake using biotin-avidin interactions, demonstrating potential for AML diagnosis and therapy (Fig. 5F) [78].

These advancements highlight the versatility of CD33-targeted approaches in both therapeutic and diagnostic applications for AML.

### CD38

CD38, also known as cyclic ADP ribose hydrolase, is a 45-kD type II transmembrane glycoprotein [156]. As an exoenzyme, it converts nicotinamide adenine dinucleotide (NAD) into adenosine diphosphate ribose (ADPR) and cyclic ADP ribose (cADPR), the latter acting as a key second messenger in Ca^2+^ signaling [157–159]. It is highly expressed in hematologic malignancies, including myeloma, chronic myeloid leukemia (CML), and AML, but shows low expression in normal tissues [160]. Experimental studies indicate that CD38 activation promotes proliferative signaling, whereas anti-CD38 antibodies inhibit its enzymatic activity and suppress cell growth [156]. Daratumumab, an anti-CD38 antibodies approved by FDA for treatment of multiple myeloma (MM) [161, 162], has recently been applied in NDDS both as a therapeutic agent and as a targeting ligand for AML nanomedicines.

**Fig. 6:**
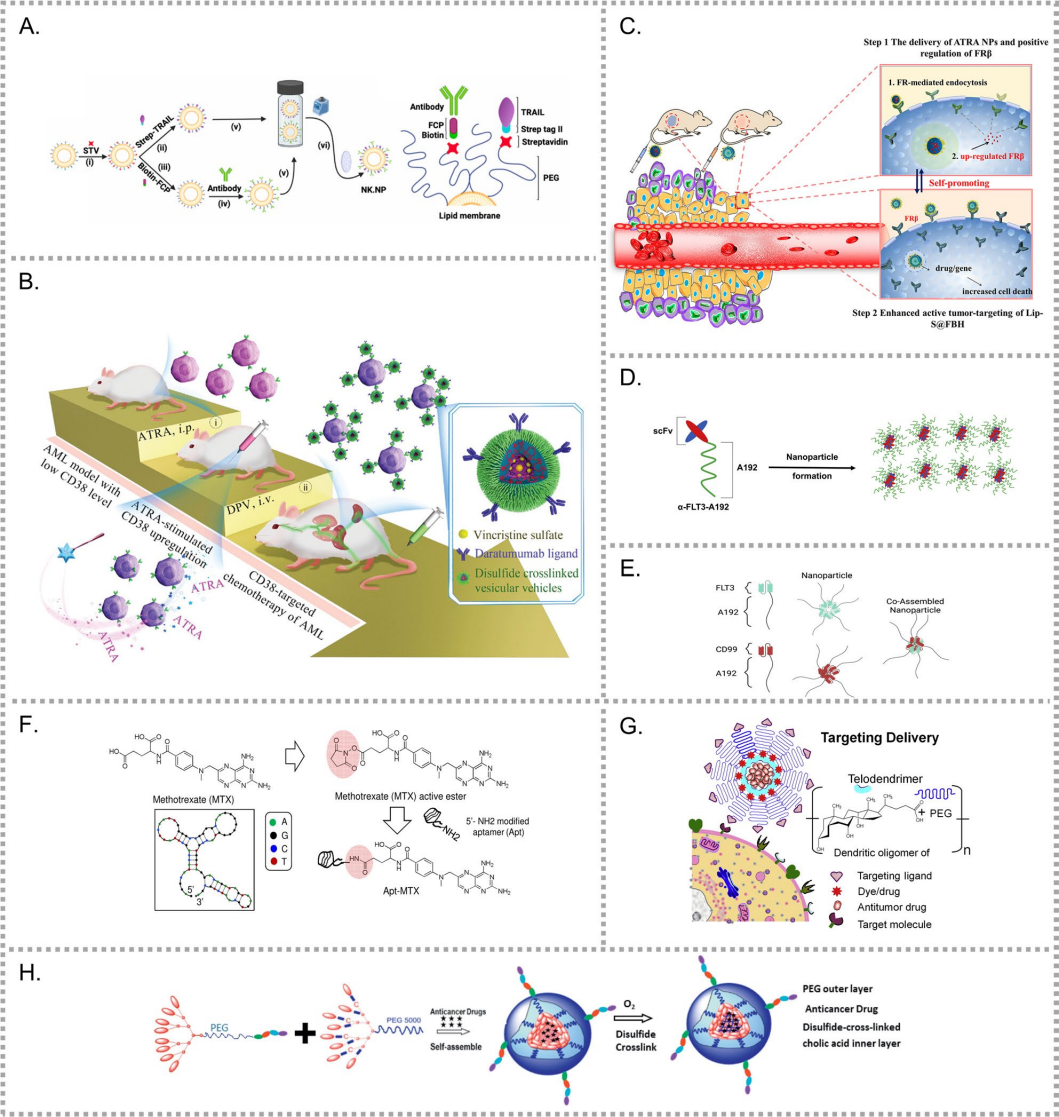
Other targeted nanomedicines for leukemia cells. (**A**) NK·NPs co-displaying TRAIL and daratumumab mimic NK cells for CD38-targeted AML therapy with enhanced cytotoxicity [79]. (**B**) ATRA combined with daratumumab-decorated polymersomal vincristine (DPV) induces CD38 upregulation and improves therapeutic efficacy in CD38-low AML [80]. (**C**) ATRA-loaded albumin NPs enhance FR-β expression and improve targeting efficiency of FA-siRNA nanocarriers in AML [84]. (**D**) Anti-FLT3 scFv fused with A192 polypeptide enables stable and cost-effective FLT3-targeted delivery [88]. (**E**) Dual-targeting NPs co-assembled from CD99–A192 and FLT3–A192 fusion proteins enhance AML therapy via CD99 and FLT3 recognition [89]. (**F**) CD117-specific ssDNA aptamer-drug conjugate (apt-MTX) enables targeted methotrexate (MTX) delivery with enhanced intracellular accumulation and reduced systemic toxicity [92]. (**G**) CLL1-L1 peptide-functionalized telodendrimer micelles enable selective DNR delivery to CLL-1^+^ LSCs [93]. (**H**) disulfide-crosslinked CLL1-targeted micelles [94]

A research team in Ireland conjugated Daratumumab to PEG-lysed liposomes, which were functionalized with recombinant human TNF-related apoptosis-inducing ligand (TRAIL) (Fig. 6A) [79]. This approach was inspired by the finding that soluble TRAIL showed limited cytotoxicity in clinical trials, while membrane-bound TRAIL on NK cells exhibited potent antitumor activity. The NK·NPs maintained strong tumoricidal effects and avoided inactivation by tumor-derived immunosuppressive factors. The inclusion of daratumumab also enabled active CD38 targeting.

Given the heterogenous expressive of CD38 level in AML patients, CD38-targeted nanomedicine may face translational challenges. To overcome this limitation, researchers introduced all-trans retinoic acid (ATRA) to induce CD38 upregulation, thereby ensuring sustained molecular target. ATRA treatment significantly increased CD38 expression in AML cell lines and primary LCs, regardless of baseline levels [163, 164]. Subsequently, a daratumumab-decorated polymersomal vincristine sulfate (DPV) was developed and combined with ATRA for AML treatment in a CD38-low animal model (Fig. 6B) [80]. While DPV monotherapy produced modest benefits, the combination therapy tripled the median survival time, demonstrating the synergistic therapeutic potential of this strategy.

### Folate receptor

Human folate receptors (FR) mediate folic acid (FA) internalization [165, 166]. FR-β is expressed at low level in the placenta and hematopoietic cells [167–169]. Although it serves as a neutrophilic lineage marker, FR-β on granulocytes is incapable of binding FA due to post-translational modifications, whereas FR-β overexpressed on AML cells retains this ability [81, 170]. In addition to being a precursor for nucleic acid synthesis, FA acts as a cofactor in multiple metabolic reactions [167, 171, 172]. The high proliferative state of AML cells leads to FR-β overexpression to meet increased FA demand [173–175], making it an attractive target for drug delivery.

ATRA can upregulate FR-β expression in AML cells. Based on this mechanism, FA-modified, DOX-loaded liposomes (f-L-DOX) were developed for AML therapy [81]. f-L-DOX enhanced FR-specific DOX uptake, which was unaffected by serum folate levels, and combination with ATRA further increased cytotoxicity. These results demonstrate the scalability and clinical potential of f-L-DOX [82, 83]. This ATRA-mediated FR-β upregulation was also utilized by Wang’s team [84]. They designed an ATRA-loaded albumin nanoparticle system co-administered with FA-modified, siRNA-encapsulating albumin nanocarriers, wherein ATRA enhanced the targeting efficiency of siRNA carriers (Fig. 6C). Similarly, FA-modified liposomes were used for co-delivery of DNR and emetine (Eme) under methotrexate (MTX) promoted FR-β expression, thereby priming selective liposomal uptake [85]. Building on this strategy, lipid-bilayer-coated mesoporous silica nanoparticles (LB-MSNs) were designed to encapsulate paclitaxel (PTX) and tanshinone IIA [86]. The lipid coating effectively prevented premature drug release and reduced hemolysis by shielding the silica surface, which would interact with tetra-alkylammonium groups on erythrocyte membranes.

### FLT3

*FMS-like tyrosine kinase 3(FLT3)* is a critical oncogene in AML, encoding a membrane-bound class III receptor tyrosine kinase [176, 177]. The internal tandem duplication (ITD) mutation within its juxtamembrane domain leads to ligand-independent activation, which associates with poor prognosis [178–180]. Although *FLT3* mutations occur in approximately 30% of newly diagnosed AML cases [181, 182], aberrant FLT3 expression and activation are not restricted to FLT3-mutant AML. FLT3 overexpression has also been reported in *FLT3* wild-type AML, where it undergoes phosphorylation and remains sensitive to FLT3 kinase inhibition [183, 184]. These findings suggest that FLT3 represents a relevant biological and therapeutic target, supporting the broader applicability of FLT3-targeted drug delivery strategies.

FLT3 has been widely explored for targeted drug delivery. One strategy use FLT3 ligand (FLT3L) as a targeting ligand conjugated to poly(amidoamine) (PAMAM) dendrimers [87]. These nanocarriers were loaded with *miR-150*, a tumor-suppressive microRNA downregulated by MLL-fusion proteins and MYC/LIN28 signaling that induces FLT3 overexpression. Restoring *miR-150* expression produced strong anti-leukemic effects in vivo, and co-administration with a bromodomain inhibitor showed synergistic inhibition of FLT3-overexpressing AML.

Another targeting approach utilizes the scFv of anti-FLT3 antibodies. Park et al. fused anti-FLT3 scFv with an elastin-like polypeptide (A192), generating a stable and cost-effective nanoplatform efficiently produced in *Escherichia coli* (Fig. 6D) [88]. Their study also revealed that FLT3-ITD AML exhibited elevated CD99 expression. Based on this, dual-targeting nanoparticles, were developed by co-assembling CD99–A192 and FLT3–A192 fusion proteins, significantly enhancing therapeutic efficacy (Fig. 6E) [89].

### CD117

CD117, also known as c-KIT, is a receptor tyrosine kinase encoded by the proto-oncogene *KIT* on chromosome 4q12 [185, 186]. It is expressed in a subset of hematopoietic stem cells (HSCs), where it regulates normal hematopoiesis with expression decreasing during differentiation [187–189]. Upon binding to its ligand, stem cell factor (SCF), CD117 activates downstream signaling via tyrosine phosphorylation, promoting cell proliferation, differentiation, migration, and survival [189–192]. CD117 represents a promising therapeutic target in AML, as it is upregulated in approximately 80% of AML patients [193, 194], correlating with poor prognosis [193, 195].

To exploit this target, an ADC was developed by linking the anti-CD117 mAbs (LMJ729) to the cytotoxic maytansinoid DM1 [90]. Researchers demonstrated that non-ligand-blocking ADCs showed significantly c-KIT degradation efficiency, particularly in the presence of SCF. This effect was consistent in both wild-type and mutant c-KIT cell lines, indicating potential therapeutic value for CD117-positive AML. However, LMJ729 was reported to induce hypersensitivity reactions via Fc receptor interactions. To address this, Kim’s team developed NN2101, a highly fucosylated and galactose-deficient mAb, which was subsequently conjugated to DM1 [91].

Considering the time, cost and complexity associated with humanized antibody production and subsequent drug conjugation, an alternative approach used a CD117-specific single-stranded DNA (ssDNA) aptamer as a drug carrier [92]. The resulting Apt-MTX conjugate achieved significantly higher intracellular drug concentrations than free MTX, enabling sub-toxic dosing while maintaining therapeutic efficacy and minimizing systemic toxicity (Fig. 6F).

### CLL-1

C-type lectin-like molecule-1 (CLL-1), or C-type lectin domain family 12 member A (CLEC12A), is involved in the establishment of innate and adaptive immunity [196–198]. As a type II transmembrane glycoprotein, CLL-1 is expressed on LSCs of AML and normal myeloid cells, but it is absent in normal HSCs [199, 200]. This selective expression enables the distinction between LSCs and HSCs, making CLL-1 an attractive therapeutic target for AML.

Through phage display screening, peptides containing the LR (S/T) motif were identified for specific binding to CLL-1. Among them, CLL1-L1 was selected for further modification and conjugated to the surface of telodendrimer-based micelles (Fig. 6G) [93]. These micelles encapsulated DNR for targeted delivery to LSCs. Compared to anti-CLL-1 antibodies, CLL1-L1 peptide-functionalized micelles reduced off-target toxicity and minimized damage to normal hematopoietic cells. To enhance structural stability and prevent premature drug release, disulfide crosslinking was introduced into the micellar framework (Fig. 6H), resulting in improved therapeutic efficacy against AML and LSCs in a patient-derived xenograft (PDX) model [94].

### CD34

CD34, encoded on chromosome 1q, is a 45-kDa type I glycoprotein selectively expressed on hematopoietic progenitor cells [201–203]. CD34^+^CD38^-^ cells have been identified as LSCs or leukemia initiation cells [204, 205]. Moreover, CD34 expression correlates with poor chemotherapy response and shorter remission duration [206, 207]. To target CD34, Carrion et al. conjugated the My10 mAb onto DOX-loaded liposomes, generating CD34-specific immunoliposomes [95]. These nanocarriers selectively bound to CD34 but were not internalized by LCs, leading to localized extracellular DOX release near LCs. Notably, non-CD34-expressing cells showed a higher IC₅₀ for CD34-targeted liposomes than for free DOX, indicating enhanced selectivity and reduced systemic toxicity.

### CD99

CD99 is a 32-kDa type II transmembrane glycoprotein essential for cell migration and adhesion [208, 209]. Its overexpression has been reported in myelodysplastic syndromes (MDS) and AML [210–212], and it serves as a reliable marker for LSCs [213]. Targeting CD99 with mAbs has been reported to reduce leukemic burden in AML xenograft models [212, 214]. However, the complex production process and high cost of mAbs remain major obstacles. To overcome these limitations, scFvs have been explored as an alternative due to their comparable specificity and rapid production in *Escherichia coli*. To further enhance therapeutic efficacy, an anti-CD99 scFv was fused with elastin-like polypeptide (ELP) to form a nanoworm, which selectively targeted CD99 and exhibited a prolonged pharmacokinetic half-life [96].

### CD96

CD96, also known as Tactile, is an immunoglobulin superfamily member expressed on T cells and NK cells, where it mediates cell adhesion during the late immune response [215–217]. Subsequent studies identified that CD96 is selectively expressed on CD34^+^CD38^−^ AML cells, suggesting its potential as a therapeutic target for eliminating LSCs [213]. A study reported that approximately 14.5% of AML patients harbored CD34^+^ CD38^−^ CD96^+^ LSCs [97]. Based on this, researchers integrated targeted nanomedicine with PDT to overcome its inherent limitations, such as photosensitizer toxicity, poor targeting specificity, and limited photon penetration [97]. They synthesized PEGylated calcium phosphosilicate nanoparticles (CPSNPs) functionalized with sulfo-NHS, which subsequently conjugated with anti-CD96 antibodies for selective targeting.

## Strategies for BM-targeted drug delivery

BM is the primary hematopoietic niche where blood cells originate and mature. However, it also functions as a protective reservoir for LCs, particularly after chemotherapy, allowing minimal residual disease (MRD) to persist. The LCs within BM microenvironment often develop drug resistance through interactions with stromal components, extracellular matrix proteins, and cytokine signaling networks, leading to disease relapse [218–220]. Therefore, eliminating residual LCs in the BM is critical to improving patient outcomes.

To achieve active BM-targeted drug delivery, two primary approaches have been investigated. The first exploits the natural homing ability of HSCs to the BM. This involves functionalizing carriers with ligands that mimic the interactions between HSCs and the BM niche, or by coating NPs with HSC membranes to enhance BM accumulation. The second strategy targets the BM microenvironment itself, focusing on receptors or molecular markers that are aberrantly expressed in the BM of AML patients. By integrating these targeting strategies, BM-specific drug delivery platforms can improve therapeutic efficacy while minimizing off-target toxicity. Continued investigation of BM niche interactions and the development of innovative delivery platforms will be essential to overcoming resistance and preventing relapse. Nanocarriers designed for BM-targeted drug delivery are summarized in Table 2.

**Table 2:** Nanocarriers designed for BM targeting in AML treatment

Target	Targeting tool	Cargo	Carrier	Stimuli		Animal model			Year	Ref
						Type	Cell	Site		
HRs	HSCM	aPD-1	Biomimetic NPs		-	CDX	C1498-Luc	Disseminated	2018	[221]
HRs	AMLCM	DOX	Biomimetic NPs		-	CDX	C1498-Luc	Disseminated	2020	[222]
HRs	HSPCM	Ara-C	Biomimetic NPs		-	CDX(SRM)	Ka539/MLL-AF9-GFP transduced BM	Disseminated	2024	[223]
HRs	HSCM	aTIM-3	CR-TNG		Hypoxia	CDX	Luc-HL-60	Disseminated	2024	[224]
E-selectin	ESTA	NPs	PSP		-	-	-	-	2011	[225]
E-selectin	ESTA	PTL	MSV		-	PDX	Primary cells	Disseminated	2016	[226]
VLA-4	LDV	siRNA	LNPs		-	PDX	Primary cells	Disseminated	2023	[227]
VLA-4/CXCR4	VCAM-1/CXCL12	Cas9 RNP	MSCM-NFs		-	CDX	THP-1	Disseminated	2021	[228]
HARE	HA	SOR	Micelle			PDX	Primary cells	Disseminated	2022	[229]
HAP	ALN	Ara-C	Liposome		GSH	CDX	C1498	Disseminated	2022	[230]
TRAP	TBP	MVC	TBP-NP		-	CDX (SRM)	MLL-AF9-transduced LSK cells	Disseminated	2021	[231]

Abbreviations: HSCM, hematopoietic stem cell membrane; AMLCM, acute myeloid leukemia cell membrane; HSPCM, hematopoietic stem/progenitor cell membrane; MSV, bone marrow-targeted multistage vector; MVC, Maraviroc. Others are defined in the Abbreviations section

### Homing receptors

Hematopoietic stem/progenitor cells (HSPCs) and AML cells possess an intrinsic ability to home to the BM through a multistep process involving adhesion, rolling, and nesting. This process is mediated by homing receptors (HRs) such as CXCR4, VLA-4, VLA-5, CD44, and LFA-1, as well as selectins expressed on HSPCs or AML cells [232–234]. CXCL12 secreted by stromal cells recruits CXCR4-expressing cells to the BM sinusoids, where E-selectin and P-selectin on endothelial interact with glycoprotein ligands on cells to mediate rolling [235, 236]. Subsequent firm adhesion to the vessel wall is mediated by interactions such as VLA-4/LFA-1 – ICAM-1, CD44 – HA, and VLA-5 – fibronectin [237–239]. Finally, under the CXCL12 gradient, activated adhesion receptors such as VLA-4 and CD44 facilitate transendothelial migration into the BM [240, 241].

**Fig. 7:**
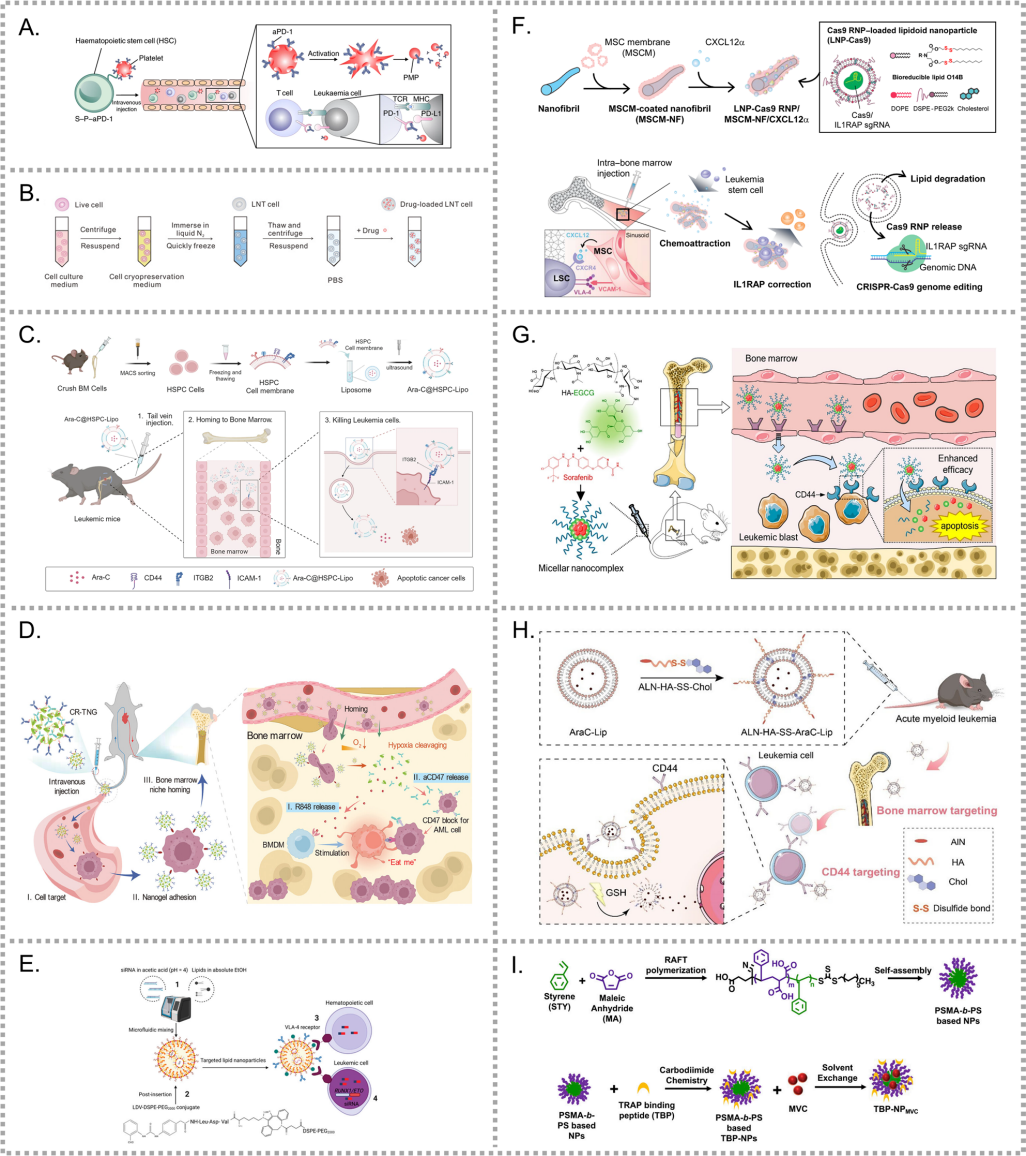
Schematic illustrations of diverse bone marrow (BM)-targeted nanocarriers for AML. (**A**) HSC membrane-platelet hybrid nanoparticles functionalized with aPD-1 for BM-targeted immune checkpoint blockade [221]. (**B**) Liquid nitrogen-treated (LNT) AML cell-derived carriers for BM-targeted doxorubicin delivery and AML vaccination [222]. (**C**) HSPC membrane-coated Ara-C-loaded liposomes enabling BM homing via adhesion molecules [223]. (**D**) Hypoxia-responsive albumin nanogels co-delivering aCD47 and R848, guided by TIM-3 targeting and BM accumulation [224]. (**E**) LDV peptide-modified lipid nanoparticles targeting VLA-4 receptor for BM-selective siRNA delivery [227]. (**F**) MSC membrane-coated nanofibrils loaded with CXCL12α and LNP-Cas9 for LSC-targeted CRISPR-Cas9 gene editing in the BM [228]. (**G**) HA-EGCG/SOR self-assembled micellar nanocomplex (sora-MNC) for BM-targeted delivery and selective uptake by CD44^+^ leukemic cells [229]. (**H**) ALN-HA-SS-AraC-Lip liposomes for hydroxyapatite-targeted BM delivery, CD44-mediated leukemic cell uptake, and redox-responsive Ara-C release [230]. (**I**) TRAP-binding peptide (TBP)-modified PSMA-b-PS micelles enable BM-targeted delivery of CCR1/5 inhibitors via TRAP-mediated endosteal accumulation [231]

By utilizing cell membranes as nanocarriers, NPs can mimic natural homing mechanisms while competitively binding adhesion molecules, thereby preventing LSCs from engaging these receptors. Hu et al. developed a biomimetic drug delivery system to transport PD-1 antibodies (aPD-1) to the BM [221]. In this system, HSC plasma membranes were covalently conjugated with aPD-1-decorated platelets through a click reaction, forming HSC-Platelet-aPD-1 (S-P-aPD-1) (Fig. 7A). S-P-aPD-1 was demonstrated enhanced BM accumulation, prolonged circulation time and reduced toxicity. Notably, it significantly improved T-cell-mediated anti-leukemia efficacy compared to the free aPD-1. However, unintended platelet activation during circulation remains a concern, as it may induce pro-inflammatory responses or systemic T-cell activation.

A research team utilized liquid nitrogen-treated (LNT) AML cells as carriers for DOX delivery (Fig. 7B) [222]. LNT treatment eliminated leukemogenic potential while retaining cellular structure and BM-homing properties. In addition to serving as an efficient drug carrier that prolonged survival of AML mice, LNT cells also acted as a cancer vaccine, inducing protective immunity against AML when administered with an adjuvant.

Li et al. extracted membranes from HSPCs and fused them with Ara-C-loaded liposomes to generate Ara-C@HSPC-Lipo (Fig. 7C) [223]. Their study demonstrated that adhesion molecules such as CD44 and ITGB2 on HSPC-derived membranes conferred BM-targeting capability.

In another study, a hypoxia-responsive albumin nanogel was designed to enhance the efficacy of immune checkpoint blockade agents, aCD47 and resiquimod (R848) [224]. CD47/R848@HSA-TIM-3 (CR-TNG) was constructed by self-assembling human serum albumin (HSA), aCD47, R848, and the hypoxia-sensitive cross-linker (NHS-AZO-NHS), followed by covalent coating with aTIM-3. After intravenous administration, aTIM-3 specifically recognized TIM-3 on circulating AML cells, facilitating CR-TNG attachment. Exploiting the BM-homing property of AML cells, CR-TNGs preferentially accumulated in the BM and underwent cleavage in response to the hypoxic microenvironment (Fig. 7D).

### E-selectin

As noted above, E-selectin (or CD62E) is specifically expressed on BM endothelial cells at tumor cell homing sites, making its ligand a promising target for BM-directed drug deliver [237, 240]. Building on this, Mann and colleagues identified an E-selectin-specific thioaptamer ligand (ESTA), and developed ESTA-functionalized porous silicon microparticles (ESTA-PSP) to modulate the biodistribution of payloads [225]. Porous silicon enables the loading of diverse cargoes, including proteins, drugs, and even nanoparticles. ESTA-PSP exhibited high nanoparticle-loading efficiency and significantly enhanced BM accumulation while reducing hepatic and splenic sequestration in vivo. Zong et al. further designed a multistage delivery system consisting of a PTL-loaded mPEG-PLA micellar core, encapsulated within an ESTA-modified porous silicon (pSi) shell [226].

### Very late antigen-4 (VLA-4)

The VLA-4 is an adhesion receptor involved in HSCs homing and cells-matrix interactions. Although nanoparticles can pass through BM sinusoids during circulation, non-targeted LNPs exhibit insufficient retention to achieve therapeutic efficacy. To address this, VLA-4 targeted nanocarriers were designed to enable selective uptake by LSCs in the BM. The tripeptide sequence Leu-Asp-Val (LDV) in fibronectin, identified as the minimal motif required for VLA-4 binding, has been utilized to direct siRNA-loaded LNPs into the BM (Fig. 7E) [227].

Additionally, BM stromal cells secrete CXCL12 to support LSC survival and inhibit their mobilization into the bloodstream [241, 242]. Ho et al. utilized this property to develop a scaffold-mediated CRISPR-Cas9 delivery system which combined nanoparticle technology with gene-editing technology [228]. In this system, Cas9/single-guide RNA ribonucleoprotein targeting IL1RAP was encapsulated within LNPs (LNP-Cas9). Nanofibrils (NFs) were coated with mesenchymal stem cell membranes (MSCMs), onto which CXCL12α was subsequently loaded to enhance LSC recruitment. LNP-Cas9 was then electrostatically assembled onto the MSCM-coated NFs, forming the final delivery construct. After injection into the BM, CXCL12α and VCAM-1 on MSCM-NFs interacted with CXCR4 and VLA-4 on LSCs, promoting internalization of the gene-editing complex (Fig. 7F).

### Hyaluronan receptor for endocytosis (HARE)

The HARE, distinct from CD44 and ICAM-1, is essential in systemic HA clearance [243, 244]. Intravenous administration of HA results in preferential accumulation in the BM [245, 246], suggesting its potential for BM-targeted drug delivery. Based on this, Bae et al. conjugated HA with the anti-leukemia agent epigallocatechin-3-O-gallate (EGCG) to form HA-EGCG. The resulting conjugate self-assembled with sorafenib (SOR) into a bone marrow-targeting micellar nanocomplex (Sora-MNC) (Fig. 7G) [229]. After reaching the BM, Sora-MNC selectively targeted leukemic blasts with high CD44 expression.

### Hydroxyapatite (HAP)

Beyond HR-based strategies, the bone mineral HAP represents another effective target for BM-directed drug delivery. Alendronate (ALN), with high affinity for HAP, been widely applied as BM-targeting ligand in models of osteoporosis [247, 248], osteosarcoma [249, 250], CML [251], MDS [252], MM [253] and bone metastasis originated from breast cancer [254, 255] or prostate cancer [256, 257].

In AML, Wu et al. developed a multifunctional liposomal system (ALN-HA-SS-AraC-Lip) that demonstrated significant anti-leukemic efficacy [230]. ALN-HA was synthesized and conjugated to cholesterol via a bio-reducible disulfide (-SS-) linker. This design enabled BM targeting, sequential recognition of CD44^+^ leukemic cells, and redox-responsive drug release within the bone microenvironment (Fig. 7H).

### Tartrate-resistant acid phosphatase (TRAP)

TRAP is a protein secreted by osteoclasts and deposited at endosteal bone resorption sites, making it a promising BM target. In leukemia, C-C chemokine ligand 3 (CCL3) plays a pivotal role in disease progression, but inhibitors of its receptors (CCR1 and CCR5) have shown limited efficacy in vivo due to poor BM accumulation [231]. To address this, poly (styrene-alt-maleic anhydride)-b-poly(styrene) (PSMA-b-PS) was used as the micellar skeleton due to its passive BM accumulation. To further enhance BM targeting, a TRAP-binding peptide (TBP) was conjugated onto the nanocarrier (Fig. 7I). TBP, a 13-amino acid oligopeptide, was identified through an M13 phage display library screening using TRAP as the target bait [258]. TBP has also been utilized to facilitate nanocarriers accumulation at bone fracture sites [259, 260], further demonstrating the confirm BM targeting ability.

## Conclusion and future directions

From the early 2000s, nanotechnology has gradually evolved from basic research to applied science, promoting the application of NDDS in medical field. Since traditional chemotherapy remains the primary treatment for AML, NDDSs have emerged as great potential in improving the antitumor efficacy of free drugs. The predominant advantages applied in AML include [261, 262]: (1) Targeted drug delivery. Active targeting can be achieved by surface modification with specific ligands or using the ligands as carrier. Which endowers classical chemotherapeutic drugs target ability without chemical modification, avoiding non-specific distribution. (2) Prolonged circulation time. Drugs encapsulated in nanocarriers are shielded from premature enzymatic degradation in the bloodstream, thereby improving their stability and extending their half-life. (3) Controlled drug release. The nanocarrier materials can be engineered to respond to specific stimuli such as pH [26], hypoxia [224], laser [28], and GSH [65], which differ between healthy and tumor tissues. (4) Crossing biological barriers. The small size of nanoparticles allows them to penetrate blood-bone marrow barrier (BMB) [226]. (5) Multi-drug delivery. NDDSs can be engineered to co-deliver multiple drugs, which is particularly advantageous in AML, where combination therapy is always required.

Despite significant progress, all the studies are still in the preclinical stage. Challenges hinder the clinical translation of NDDSs include: (1) Toxicity and biocompatibility concerns. Some nanocarrier materials, particularly metals (such as copper, iron), silicon-based nanoparticles (such as SiO₂), and high-molecular-weight polymers (such as polystyrene, polylactic acid), are difficult to degrade and may accumulate in organs, causing systemic toxicity. (2) Impaired targeting efficiency. Some tumor-targeting receptors are also expressed on normal tissues, leading to off-target effects. (3) Manufacturing challenges. The complex fabrication process of active-targeted NDDSs presents scalability and quality control issues. (4) Complex approval process and supervision standards. NDDS are still a relatively new field. The complex structure and mechanisms of action need more toxicological and clinical trials to assess safety and efficacy. And the uniform international evaluation standards of preparation and characterization of NPs is deficient.

While these limitations present considerable obstacles, they do not overshadow the promising outcomes achieved in preclinical AML models. In response to the clinical translational obstacles mentioned above, several strategies can be proposed:

Future NDDS design should prioritize biodegradable and biomimetic materials. Inspired by the concept of CAR-T therapy, which involves extracting a patient’s own T cells, genetically modifying them, and reinfusing them back into the patient [263, 264], a similar strategy may be envisioned using AML cells. AML cells could be isolated from patients, and their membranes harnessed to coat anti-tumor drugs or drug-loaded nanoparticles. Since AML is a highly heterogeneous disease, with genetic, phenotypic, and microenvironmental differences among patients, which contribute to variable responses to standard chemotherapy regimens. These membrane-camouflaged nanoparticles could then be reinfused into the same patient, enabling a highly personalized drug delivery approach that leverages tumor-derived homing capabilities while minimizing immune response.

In addition, off-target effect remains a major obstacle in targeted therapy, particularly in the late treatment stage when tumor burden decreases and MRD shielding in the BM becoming hard to target. Therefore, targeting BM is crucial. Few studies have focused on this aspect, most of them are biomimetic NDDSs, demonstrating their great potential once again. Future research should focus on maintaining membrane function during extraction and preparation to ensure stable BM-targeting ability and batch-to-batch consistency. Meanwhile, it is also essential to maintain consistent and high receptor expression across all disease stages. When leukemic cells express significantly higher receptor levels than normal cells, NDDSs are more likely to accumulate selectively in LCs, reducing off-target effect. As presented in this review, combining NDDS with chemotherapeutic agents that upregulate receptor expression will enhance the specificity and efficacy of targeted NDDSs. Such as MTX upregulate FR expression [85], and ATRA increases FR [81, 84] and CD38 [163, 164]. This strategy shows clinical promise in reducing off-target toxicity and overcoming limitations caused by variable receptor expression, while expanding the therapeutic potential of these drugs.

Further, LSCs represent a rare subpopulation of leukemic cells with self-renewal capacity and resistance to conventional therapies [265, 266]. They mainly reside in protective BM niches, where the microenvironment provides anti-apoptotic and quiescence-maintaining signals. This shelter enables LSCs to evade immune surveillance and survive chemotherapy, leading to relapse [218, 267]. In addition, most chemotherapeutic agents act primarily on rapidly dividing cells, quiescent LSCs can escape cytotoxic damage. Consequently, NDDSs offer a promising strategy for eliminate LSCs by selectively targeting their specific surface markers. Targeted delivery strategies against CD34, CD123, CLL1, CD96, and CD99 have shown encouraging results, with CD123-targeted NDDSs being the most extensively studied. Considering that the BM niche supports LSCs survival and can be remodeled by LSCs, dual-targeting NDDSs that combine BM-homing mechanisms with LSC-specific ligands represent an appealing strategy. Incorporating stimuli-responsive components allows these systems to release drugs precisely in response to features of the LSC niche, such as hypoxia [268] and overexpression of matrix metalloproteinases (MMPs) [269]. This approach holds promise for improving the precision and efficacy of AML treatment in the future.

Finally, for clinical translation and large-scale production, NDDS design should be simplified. Complicated synthesis and preparation processes will impair batch consistency and increase cost. And ligands should be proven efficacy, and easy to synthesis or naturally available. Future research should focus on improving targeting precision, delivery efficiency, and in vivo validation of these systems in clinically relevant AML models. In summary, NDDSs represents not only a critical future trend but also a potential breakthrough in the treatment of AML.

## Data Availability

Not applicable.

## Abbreviations

6-MP: 6-Mercaptopurine
ADC: Antibody-drug conjugate
ALN: Alendronate
AML: Acute myeloid leukemia
aPD-1: PD-1 antibodies
Ara-C: Cytarabine
ASOs: Antisense oligodeoxynucleotides
ATO: Arsenic trioxide
ATRA: All-trans retinoic acid
BM: Bone marrow
CLL-1: C-type lectin-like molecule-1
Cur: Curcumin
CXCR4: C-X-C chemokine receptor 4
DNR: Daunorubicin
DOX: Doxorubicin
EDS: Edelfosine
Eme: Emetine
EPR: Enhanced permeation and retention
FA: Folic acid
FH: Feraheme
FLT3: FMS-like tyrosine kinase 3
FR: Folate receptors
GA: Gallic acid
GDH: Graphene quantum dots
GEM: Genetically engineered mouse
GSH: Glutathione
HA: Hyaluronic acid
HAP: Hydroxyapatite
HARE: Hyaluronan receptor for endocytosis
HDC: Histamine dihydrochloride
HMPB: Hollow mesoporous Prussian blue
HRs: Homing receptors
HSCs: Hematopoietic stem cells
HSPCs: Hematopoietic stem/progenitor cells
HSCT: Hematopoietic stem cell transplantation
HumF: Humanized Archaeoglobus ferritin
ICG: Indocyanine green
ITD: Internal tandem duplication
LC: Leukemia cells
LDV: Leu-Asp-Val
LNPs: Lipid nanoparticles
LONp: Lanthanide oxyfluoride nanoparticle
LPPs: Lipopolyplex nanoparticles
LSCs: Leukemia stem cells
Lut: Luteolin
mAbs: Monoclonal antibodies
MDS: Myelodysplastic syndromes
MM: Multiple myeloma
MRD: Minimal residual disease
MTX: Methotrexate
NCs: Nanoconjugates
NDDSs: Nano-drug delivery systems
NP: Nanoparticles
PB: Peripheral blood
PBNPs: Prussian blue nanoparticles
PDT: Photodynamic therapy
PDX: Patient-derived xenograft
PTX: Paclitaxel
PPNPs: Porous silicon microparticles
PSP: Porous silicon microparticles
PTL: Parthenolide
PTX: Paclitaxel
RNR: Ribonucleotide reductase
ROS: Reactive oxygen species
scFv: Single-chain variable fragment
siRNA: Small interference RNA
SOR: Sorafenib
TBP: TRAP-binding peptide
TDT: Targeted drug train
Tf: Transferrin
TRAIL: TNF-related apoptosis-inducing ligand
TRAP: Tartrate-resistant acid phosphatase
VEN: Venetoclax
VLA-4: Very late antigen-4

## References

[1] Döhner H, Wei AH, Appelbaum FR, Craddock C, DiNardo CD, Dombret H, et al. Diagnosis and management of AML in adults: 2022 recommendations from an international expert panel on behalf of the ELN. Blood. 2022;140(12):1345–77. 35797463 10.1182/blood.2022016867

[2] Bataller A, DiNardo CD, Bazinet A, Daver NG, Maiti A, Borthakur G, et al. Targetable genetic abnormalities in patients with acute myeloblastic leukemia across age groups. Am J Hematol. 2024;99(4):792–96. 38361282 10.1002/ajh.27236

[3] Hoff FW, Huang Y, Welkie RL, Swords RT, Traer E, Stein EM, et al. Molecular characterization of newly diagnosed acute myeloid leukemia patients aged 60 years or older: a report from the beat AML clinical trial. Blood Cancer J. 2025;15(1):55. 40180903 10.1038/s41408-025-01258-0 PMC11968959

[4] Matthews AH, Perl AE, Luger SM, Gill SI, Lai C, Porter DL, et al. Real-world effectiveness of intensive chemotherapy with 7+3 versus venetoclax and hypomethylating agent in acute myeloid leukemia. Am J Hematol. 2023;98(8):1254–64. 37334852 10.1002/ajh.26991 PMC11057024

[5] Röllig C, Steffen B, Schliemann C, Mikesch JH, Alakel N, Herbst R, et al. Single or Double induction with 7 + 3 containing standard or high-dose daunorubicin for newly diagnosed AML: the randomized DaunoDouble trial by the study alliance leukemia. J Clin Oncol. 2025;43(1):65–74. 39284116 10.1200/JCO.24.00235

[6] Pratz KW, Cherry M, Altman JK, Cooper BW, Podoltsev NA, Cruz JC, et al. Gilteritinib in combination with induction and consolidation chemotherapy and as maintenance therapy: a phase IB study in patients with newly diagnosed AML. J Clin Oncol. 2023;41(26):4236–46. 37379495 10.1200/JCO.22.02721

[7] Joudinaud R, Boudry A, Fenwarth L, Geffroy S, Salson M, Dombret H, et al. Midostaurin shapes macroclonal and microclonal evolution of FLT3-mutated acute myeloid leukemia. Blood Adv. 2025;9(2):365–74. 39418643 10.1182/bloodadvances.2024014672 PMC11787458

[8] Erba HP, Montesinos P, Kim HJ, Patkowska E, Vrhovac R, Žák P, et al. Quizartinib plus chemotherapy in newly diagnosed patients with FLT3-internal-tandem-duplication-positive acute myeloid leukaemia (QuANTUM-First): a randomised, double-blind, placebo-controlled, phase 3 trial. Lancet. 2023;401(10388):1571–83. 37116523 10.1016/S0140-6736(23)00464-6

[9] Ivosidenib boosts OS with azacitidine in AML. Cancer Discov. 2022;12(7):1602–03. 10.1158/2159-8290.CD-NB2022-0035 35532213

[10] de Botton S, Montesinos P, Schuh AC, Papayannidis C, Vyas P, Wei AH, et al. Enasidenib vs conventional care in older patients with late-stage mutant-IDH2 relapsed/refractory AML: a randomized phase 3 trial. Blood. 2023;141(2):156–67. 35714312 10.1182/blood.2021014901 PMC10644040

[11] Gangat N, Tefferi A. Venetoclax schedule in AML: 7 vs 14 vs 21 vs 28 days. Blood Cancer J. 2025;15(1):56. 40180902 10.1038/s41408-025-01270-4 PMC11968881

[12] Wang M, Chen S, Zhang Q, Yuan L, Wang X, Zhang J, et al. Comparison of autologous hematopoietic cell transplantation, matched sibling donor hematopoietic cell transplantation, and chemotherapy in patients with favorable- and intermediate-risk acute myeloid leukemia. Front Immunol. 2024;15:1511057. 39845970 10.3389/fimmu.2024.1511057 PMC11751218

[13] Mei D, Xue Z, Zhang T, Yang Y, Jin L, Yu Q, et al. Immune isolation-enabled nanoencapsulation of donor T cells: a promising strategy for mitigating GVHD and treating AML in preclinical models. J Immunother Cancer. 2024;12(9). 10.1136/jitc-2023-008663 PMC11381671 39242117

[14] Freyer CW, Porter DL. Cytokine release syndrome and neurotoxicity following CAR T-cell therapy for hematologic malignancies. J Allergy Clin Immunol. 2020;146(5):940–48. 32771558 10.1016/j.jaci.2020.07.025

[15] Wang X, Zhang Y, Xue S. Recent progress in chimeric antigen receptor therapy for acute myeloid leukemia. Ann Hematol. 2024;103(6):1843–57. 38381173 10.1007/s00277-023-05601-y

[16] Aalhate M, Mahajan S, Dhuri A, Singh PK. Biohybrid nano-platforms manifesting effective cancer therapy: fabrication, characterization, challenges and clinical perspective. Adv Colloid Interface Sci. 2025;335:103331. 39522420 10.1016/j.cis.2024.103331

[17] Kong X, Xie X, Wu J, Wang X, Zhang W, Wang S, et al. Combating cancer immunotherapy resistance: a nano-medicine perspective. Cancer Commun (Lond). 2025. 10.1002/cac2.70025 PMC12328096 40207650

[18] Zou L, Xian P, Pu Q, Song Y, Ni S, Chen L, et al. Nano-drug delivery strategies affecting cancer-associated fibroblasts to reduce tumor metastasis. Acta Pharm Sin B. 2025;15(4):1841–68. 40486841 10.1016/j.apsb.2025.02.040 PMC12138110

[19] Cortes JE, Lin TL, Asubonteng K, Faderl S, Lancet JE, Prebet T. Efficacy and safety of CPX-351 versus 7 + 3 chemotherapy by European LeukemiaNet 2017 risk subgroups in older adults with newly diagnosed, high-risk/secondary AML: post hoc analysis of a randomized, phase 3 trial. J Hematol Oncol. 2022;15(1):155. 36289532 10.1186/s13045-022-01361-w PMC9598030

[20] Renga G, Nunzi E, Stincardini C, Pariano M, Puccetti M, Pieraccini G, et al. CPX-351 exploits the gut microbiota to promote mucosal barrier function, colonization resistance, and immune homeostasis. Blood. 2024;143(16):1628–45. 38227935 10.1182/blood.2023021380

[21] Taurin S, Nehoff H, Greish K. Anticancer nanomedicine and tumor vascular permeability; where is the missing link? J Control Release. 2012;164(3):265–75. 22800576 10.1016/j.jconrel.2012.07.013

[22] Khademi R, Mohammadi Z, Khademi R, Saghazadeh A, Rezaei N. Nanotechnology-based diagnostics and therapeutics in acute lymphoblastic leukemia: a systematic review of preclinical studies. Nanoscale Adv. 2023;5(3):571–95. 36756502 10.1039/d2na00483f PMC9890594

[23] Lim WS, Tardi PG, Xie X, Fan M, Huang R, Ciofani T, et al. Schedule- and dose-dependency of CPX-351, a synergistic fixed ratio cytarabine: daunorubicin formulation, in consolidation treatment against human leukemia xenografts. Leuk Lymphoma. 2010;51(8):1536–42. 20528246 10.3109/10428194.2010.490312

[24] Meng J, Ge Y, Xing H, Wei H, Xu S, Liu J, et al. Synthetic CXCR4 Antagonistic peptide assembling with nanoscaled micelles combat acute myeloid leukemia. Small. 2020;16(31):e2001890. 32608185 10.1002/smll.202001890

[25] Zhang M, Ge Y, Xu S, Fang X, Meng J, Yu L, et al. Nanomicelles co-loading CXCR4 antagonist and doxorubicin combat the refractory acute myeloid leukemia. Pharmacol Res. 2022;185:106503. 36241000 10.1016/j.phrs.2022.106503

[26] Kong F, Bai HY, Ma M, Wang C, Xu HY, Gu N, et al. Fe_3_O_4_@Pt nanozymes combining with CXCR4 antagonists to synergistically treat acute myeloid leukemia. Nano Today. 2021;37.

[27] Bai HY, Wang T, Kong F, Zhang MC, Li ZX, Zhuang LL, et al. CXCR4 and CD44 dual-targeted Prussian blue nanosystem with daunorubicin loaded for acute myeloid leukemia therapy. Chem Eng J. 2021;405.

[28] Bai H, Sun Q, Kong F, Dong H, Ma M, Liu F, et al. Zwitterion-functionalized hollow mesoporous Prussian blue nanoparticles for targeted and synergetic chemo-photothermal treatment of acute myeloid leukemia. J Mater Chem B. 2021;9(26):5245–54. 34095945 10.1039/d1tb00548k

[29] Wang X, Wang X, Su J, Wang D, Feng W, Wang X, et al. A dual-function LipoAraN-E5 Coloaded with N(4)-myristyloxycarbonyl-1-β-d-arabinofuranosylcytosine (AraN) and a CXCR4 antagonistic peptide (E5) for blocking the dissemination of acute myeloid leukemia. ACS Nano. 2024;18(41):27917–32. 39364559 10.1021/acsnano.4c05079

[30] Díaz R, Pallarès V, Cano-Garrido O, Serna N, Sánchez-García L, Falgàs A, et al. Selective CXCR4^+^ cancer cell targeting and potent antineoplastic effect by a nanostructured version of recombinant ricin. Small. 2018;14(26). 10.1002/smll.201800665 29845742

[31] Pallarès V, Unzueta U, Falgàs A, Sánchez-García L, Serna N, Gallardo A, et al. An auristatin nanoconjugate targeting CXCR4+ leukemic cells blocks acute myeloid leukemia dissemination. J Hematol Oncol. 2020;13(1):36. 32295630 10.1186/s13045-020-00863-9 PMC7160905

[32] Pallarès V, Unzueta U, Falgàs A, Aviñó A, Núñez Y, García-León A, et al. A multivalent Ara-C-prodrug nanoconjugate achieves selective ablation of leukemic cells in an acute myeloid leukemia mouse model. Biomaterials. 2022;280:121258. 34847435 10.1016/j.biomaterials.2021.121258

[33] Pallarès V, Núñez Y, Sánchez-García L, Falgàs A, Serna N, Unzueta U, et al. Antineoplastic effect of a diphtheria toxin-based nanoparticle targeting acute myeloid leukemia cells overexpressing CXCR4. J Control Release. 2021;335:117–29. 34004204 10.1016/j.jconrel.2021.05.014

[34] Núñez Y, Garcia-León A, Falgàs A, Serna N, Sánchez-García L, Garrido A, et al. T22-PE24-H6 nanotoxin selectively kills CXCR4-high expressing AML patient cells in vitro and potently blocks dissemination in vivo. Pharmaceutics. 2023;15(3). 10.3390/pharmaceutics15030727 PMC10054149 36986589

[35] Serna N, Pallarès V, Unzueta U, Garcia-Leon A, Voltà-Durán E, Sánchez-Chardi A, et al. Engineering non-antibody human proteins as efficient scaffolds for selective, receptor-targeted drug delivery. J Control Release. 2022;343:277–87. 35051493 10.1016/j.jconrel.2022.01.017

[36] Ren XH, Xu C, Li LL, Zuo Y, Han D, He XY, et al. A targeting delivery system for effective genome editing in leukemia cells to reverse malignancy. J Control Release. 2022;343:645–56. 35157940 10.1016/j.jconrel.2022.02.012

[37] Yang J, Zhang P, Mao Y, Chen R, Cheng R, Li J, et al. CXCR4-mediated codelivery of FLT3 and BCL-2 inhibitors for enhanced targeted combination therapy of FLT3-ITD acute myeloid leukemia. Biomacromolecules. 2024;25(7):4569–80. 38869359 10.1021/acs.biomac.4c00561

[38] Wang Y, Xie Y, Williams J, Hang Y, Richter L, Becker M, et al. Use of polymeric CXCR4 inhibitors as siRNA delivery vehicles for the treatment of acute myeloid leukemia. Cancer Gene Ther. 2020;27(1–2):45–55. 31028289 10.1038/s41417-019-0095-9

[39] Kong F, He H, Bai H, Yang F, Ma M, Gu N, et al. A biomimetic nanocomposite with enzyme-like activities and CXCR4 antagonism efficiently enhances the therapeutic efficacy of acute myeloid leukemia. Bioact Mater. 2022;18:526–38. 35415298 10.1016/j.bioactmat.2022.03.022 PMC8976099

[40] Li QQ, Wang F, Bai HY, Cui Y, Ma M, Zhang Y. Hollow mesoporous Prussian blue nanoparticles loaded with daunorubicin for acute myeloid leukemia treatment. ACS Appl Nano Mater. 2023;6(23):22128–41.

[41] Zhang Y, Chen L, Fu T, Xu AB, Li KQ, Hao K, et al. Self-stimulated photodynamic nanoreactor in combination with CXCR4 antagonists for antileukemia therapy. ACS Appl Mater Interfaces. 2024;16(17):21610–22. 38647446 10.1021/acsami.4c01603

[42] Citro G, Perrotti D, Cucco C, D’Agnano I, Sacchi A, Zupi G, et al. Inhibition of leukemia cell proliferation by receptor-mediated uptake of c-myb antisense oligodeoxynucleotides. Proc Natl Acad Sci USA. 1992;89(15):7031–35. 1495997 10.1073/pnas.89.15.7031 PMC49639

[43] Jin Y, Liu S, Yu B, Golan S, Koh CG, Yang J, et al. Targeted delivery of antisense oligodeoxynucleotide by transferrin conjugated pH-sensitive lipopolyplex nanoparticles: a novel oligonucleotide-based therapeutic strategy in acute myeloid leukemia. Mol Pharm. 2010;7(1):196–206. 19852511 10.1021/mp900205r PMC4342499

[44] Huang X, Schwind S, Yu B, Santhanam R, Wang H, Hoellerbauer P, et al. Targeted delivery of microRNA-29b by transferrin-conjugated anionic lipopolyplex nanoparticles: a novel therapeutic strategy in acute myeloid leukemia. Clin Cancer Res. 2013;19(9):2355–67. 23493348 10.1158/1078-0432.CCR-12-3191 PMC3644023

[45] Yang Z, Yu B, Zhu J, Huang X, Xie J, Xu S, et al. A microfluidic method to synthesize transferrin-lipid nanoparticles loaded with siRNA LOR-1284 for therapy of acute myeloid leukemia. Nanoscale. 2014;6(16):9742–51. 25003978 10.1039/c4nr01510j PMC4312591

[46] Zhu B, Zhang H, Yu L. Novel transferrin modified and doxorubicin loaded Pluronic 85/lipid-polymeric nanoparticles for the treatment of leukemia: in vitro and in vivo therapeutic effect evaluation. Biomed Pharmacother. 2017;86:547–54. 28024291 10.1016/j.biopha.2016.11.121

[47] Sun Y, Sun ZL. Transferrin-conjugated polymeric nanomedicine to enhance the anticancer efficacy of edelfosine in acute myeloid leukemia. Biomed Pharmacother. 2016;83:51–57. 27470549 10.1016/j.biopha.2016.05.046

[48] Zhu Y, Zhang W, Chen J. Binary nanodrug-delivery system designed for leukemia therapy: aptamer- and transferrin-codecorated daunorubicin- and luteolin-coloaded nanoparticles. Drug Des Devel Ther. 2023;17:1–13. 36636745 10.2147/DDDT.S387246 PMC9830956

[49] Wu W, Li Y, Liu Q, Liu T, Zhao Y, Shao H, et al. Dual-targeted drug delivery to myeloid leukemia cells via complement- and transferrin-based protein Corona. Nano Lett. 2025;25(1):147–56. 39694635 10.1021/acs.nanolett.4c04429

[50] Macone A, Masciarelli S, Palombarini F, Quaglio D, Boffi A, Trabuco MC, et al. Ferritin nanovehicle for targeted delivery of cytochrome C to cancer cells. Sci Rep. 2019;9(1):11749. 31409839 10.1038/s41598-019-48037-z PMC6692331

[51] Palombarini F, Masciarelli S, Incocciati A, Liccardo F, Di Fabio E, Iazzetti A, et al. Self-assembling ferritin-dendrimer nanoparticles for targeted delivery of nucleic acids to myeloid leukemia cells. J Nanobiotechnol. 2021;19(1):172. 10.1186/s12951-021-00921-5 PMC8190868 34107976

[52] Wang C, Zhang W, He Y, Gao Z, Liu L, Yu S, et al. Ferritin-based targeted delivery of arsenic to diverse leukaemia types confers strong anti-leukaemia therapeutic effects. Nat Nanotechnol. 2021;16(12):1413–23. 34697490 10.1038/s41565-021-00980-7

[53] Wu X, Jiao Z, Zhang J, Li F, Li Y. Expression of TFRC helps to improve the antineoplastic effect of Ara-C on AML cells through a targeted delivery carrier. J Nanobiotechnol. 2023;21(1):126. 10.1186/s12951-023-01881-8 PMC10088114 37041636

[54] Rajabinejad M, Valadan R, Tehrani M, Najafi A, Negarandeh R, Saeedi M, et al. Effective delivery of anti-PD-L1 siRNA with human heavy chain ferritin (HFn) in acute myeloid leukemia cell lines. Med Oncol. 2024;41(6):149. 38739199 10.1007/s12032-024-02393-7

[55] Suzuki S, Inoue K, Hongoh A, Hashimoto Y, Yamazoe Y. Modulation of doxorubicin resistance in a doxorubicin-resistant human leukaemia cell by an immunoliposome targeting transferring receptor. Br J Cancer. 1997;76(1):83–89. 9218737 10.1038/bjc.1997.340 PMC2223806

[56] Xu S, Zhang M, Fang X, Meng J, Xing H, Yan D, et al. A novel CD123-targeted therapeutic peptide loaded by micellar delivery system combats refractory acute myeloid leukemia. J Hematol Oncol. 2021;14(1):193. 34774070 10.1186/s13045-021-01206-y PMC8590286

[57] Xu S, Zhang M, Fang X, Hu X, Xing H, Yang Y, et al. CD123 Antagonistic peptides assembled with nanomicelles act as monotherapeutics to combat refractory acute myeloid leukemia. ACS Appl Mater Interfaces. 2022;14(34):38584–93. 35977045 10.1021/acsami.2c11538

[58] Wang Y, Liu F, Wang Q, Xiang H, Jin H, Li H, et al. A novel immunoliposome mediated by CD123 antibody targeting to acute myeloid leukemia cells. Int J Pharm. 2017;529(1–2):531–42. 28583331 10.1016/j.ijpharm.2017.06.003

[59] Liu FR, Jin H, Wang Y, Chen C, Li M, Mao SJ, et al. Anti-CD123 antibody-modified niosomes for targeted delivery of daunorubicin against acute myeloid leukemia. Drug Deliv. 2017;24(1):882–90. 28574300 10.1080/10717544.2017.1333170 PMC8244627

[60] Sun S, Zou H, Li L, Liu Q, Ding N, Zeng L, et al. CD123/CD33 dual-antibody modified liposomes effectively target acute myeloid leukemia cells and reduce antigen-negative escape. Int J Pharm. 2019;568:118518. 31319147 10.1016/j.ijpharm.2019.118518

[61] Guo J, Russell EG, Darcy R, Cotter TG, McKenna SL, Cahill MR, et al. Antibody-targeted cyclodextrin-based nanoparticles for siRNA delivery in the treatment of acute myeloid leukemia: physicochemical characteristics, in vitro mechanistic studies, and ex vivo patient derived therapeutic efficacy. Mol Pharm. 2017;14(3):940–52. 28146632 10.1021/acs.molpharmaceut.6b01150

[62] Wu H, Wang M, Dai B, Zhang Y, Yang Y, Li Q, et al. Novel CD123-aptamer-originated targeted drug trains for selectively delivering cytotoxic agent to tumor cells in acute myeloid leukemia theranostics. Drug Deliv. 2017;24(1):1216–29. 28845698 10.1080/10717544.2017.1367976 PMC8241133

[63] Wu H, Zhang L, Zhu Z, Ding C, Chen S, Liu R, et al. Novel CD123 polyaptamer hydrogel edited by Cas9/sgRNA for AML-targeted therapy. Drug Deliv. 2021;28(1):1166–78. 34121564 10.1080/10717544.2021.1934191 PMC8205012

[64] Sun D, Zhou JK, Zhao L, Zheng ZY, Li J, Pu W, et al. Novel curcumin liposome modified with hyaluronan targeting CD44 plays an anti-leukemic role in acute myeloid leukemia in vitro and in vivo. ACS Appl Mater Interfaces. 2017;9(20):16857–68. 28489348 10.1021/acsami.7b02863

[65] Qiu J, Cheng R, Zhang J, Sun H, Deng C, Meng F, et al. Glutathione-sensitive hyaluronic acid-mercaptopurine prodrug linked via carbonyl vinyl sulfide: a robust and CD44-targeted nanomedicine for leukemia. Biomacromolecules. 2017;18(10):3207–14. 28835099 10.1021/acs.biomac.7b00846

[66] Zhong Y, Meng F, Deng C, Mao X, Zhong Z. Targeted inhibition of human hematological cancers in vivo by doxorubicin encapsulated in smart lipoic acid-crosslinked hyaluronic acid nanoparticles. Drug Deliv. 2017;24(1):1482–90. 28958164 10.1080/10717544.2017.1384864 PMC8240992

[67] Cherukula K, Nurunnabi M, Jeong YY, Lee YK, Park IK. A targeted graphene nanoplatform carrying histamine dihydrochloride for effective inhibition of leukemia-induced immunosuppression. J Biomater Sci Polym Ed. 2018;29(7–9):734–49. 28994338 10.1080/09205063.2017.1390382

[68] Shao Y, Luo W, Guo Q, Li X, Zhang Q, Li J. In vitro and in vivo effect of hyaluronic acid modified, doxorubicin and gallic acid co-delivered lipid-polymeric hybrid nano-system for leukemia therapy. Drug Des Devel Ther. 2019;13:2043–55. 31388296 10.2147/DDDT.S202818 PMC6607984

[69] Darwish NHE, Sudha T, Godugu K, Bharali DJ, Elbaz O, El-Ghaffar HAA, et al. Novel targeted nano-parthenolide molecule against NF-kB in acute myeloid leukemia. Molecules (Basel, Switzerland). 2019;24(11). 10.3390/molecules24112103 PMC6600366 31163672

[70] Rothdiener M, Müller D, Castro PG, Scholz A, Schwemmlein M, Fey G, et al. Targeted delivery of SiRNA to CD33-positive tumor cells with liposomal carrier systems. J Control Release. 2010;144(2):251–58. 20184933 10.1016/j.jconrel.2010.02.020

[71] Niu F, Yan J, Ma B, Li S, Shao Y, He P, et al. Lanthanide-doped nanoparticles conjugated with an anti-CD33 antibody and a p53-activating peptide for acute myeloid leukemia therapy. Biomaterials. 2018;167:132–42. 29571049 10.1016/j.biomaterials.2018.03.025 PMC5889738

[72] Simard P, Leroux JC. pH-sensitive immunoliposomes specific to the CD33 cell surface antigen of leukemic cells. Int J Pharm. 2009;381(2):86–96. 19446624 10.1016/j.ijpharm.2009.05.013

[73] Simard P, Leroux JC. In vivo evaluation of pH-sensitive polymer-based immunoliposomes targeting the CD33 antigen. Mol Pharm. 2010;7(4):1098–107. 20476756 10.1021/mp900261m

[74] Li H, Xu S, Quan J, Yung BC, Pang J, Zhou C, et al. CD33-targeted lipid nanoparticles (aCd33lns) for therapeutic delivery of GTI-2040 to acute myelogenous leukemia. Mol Pharm. 2015;12(6):2010–18. 25871632 10.1021/mp5008212 PMC4962870

[75] Yan C, Gu J, Zhang Y, Ma K, Lee RJ. Efficient delivery of the bcl-2 antisense oligonucleotide G3139 via nucleus-targeted aCD33-NKSN nanoparticles. Int J Pharm. 2022;625:122074. 35932928 10.1016/j.ijpharm.2022.122074

[76] Zaimy MA, Jebali A, Bazrafshan B, Mehrtashfar S, Shabani S, Tavakoli A, et al. Coinhibition of overexpressed genes in acute myeloid leukemia subtype M2 by gold nanoparticles functionalized with five antisense oligonucleotides and one anti-CD33(+)/CD34(+) aptamer. Cancer Gene Ther. 2016;23(9):315–20. 27514505 10.1038/cgt.2016.33

[77] Chen H, Jayasinghe MK, Yeo EYM, Wu Z, Pirisinu M, Usman WM, et al. CD33-targeting extracellular vesicles deliver antisense oligonucleotides against FLT3-ITD and miR-125b for specific treatment of acute myeloid leukaemia. Cell Prolif. 2022;55(9):e13255. 35851970 10.1111/cpr.13255 PMC9436904

[78] Štefík P, Annušová A, Lakatoš B, Elefantová K, Čepcová L, Hofbauerová M, et al. Targeting acute myeloid leukemia cells by CD33 receptor-specific MoS(2)-based nanoconjugates. Biomed Mater. 2021;16(5). 10.1088/1748-605X/ac15b1 34280914

[79] Alizadeh Zeinabad H, Yeoh WJ, Arif M, Lomora M, Banz Y, Riether C, et al. Natural killer cell-mimic nanoparticles can actively target and kill acute myeloid leukemia cells. Biomaterials. 2023;298:122126. 37094524 10.1016/j.biomaterials.2023.122126

[80] Yue S, An J, Zhang Y, Li J, Zhao C, Liu J, et al. Exogenous antigen upregulation empowers antibody targeted nanochemotherapy of leukemia. Adv Mater. 2023;35(32):e2209984. 37321606 10.1002/adma.202209984

[81] Pan XQ, Zheng X, Shi G, Wang H, Ratnam M, Lee RJ. Strategy for the treatment of acute myelogenous leukemia based on folate receptor beta-targeted liposomal doxorubicin combined with receptor induction using all-trans retinoic acid. Blood. 2002;100(2):594–602. 12091353 10.1182/blood.v100.2.594

[82] Lu Y, Wu J, Wu J, Gonit M, Yang X, Lee A, et al. Role of formulation composition in folate receptor-targeted liposomal doxorubicin delivery to acute myelogenous leukemia cells. Mol Pharm. 2007;4(5):707–12. 17708654 10.1021/mp070058l

[83] Li H, Lu Y, Piao L, Wu J, Liu S, Marcucci G, et al. Targeting human clonogenic acute myelogenous leukemia cells via folate conjugated liposomes combined with receptor modulation by all-trans retinoic acid. Int J Pharm. 2010;402(1–2):57–63. 20883757 10.1016/j.ijpharm.2010.09.019 PMC2982872

[84] Wang D, Li H, Chen W, Yang H, Liu Y, You B, et al. Efficient tumor-targeting delivery of siRNA via folate-receptor mediated biomimetic albumin nanoparticles enhanced by all-trans retinoic acid. Mater Sci Eng C Mater Biol Appl. 2021;119:111583. 33321629 10.1016/j.msec.2020.111583

[85] Myhren L, Nilssen IM, Nicolas V, Døskeland SO, Barratt G, Herfindal L. Efficacy of multi-functional liposomes containing daunorubicin and emetine for treatment of acute myeloid leukaemia. Eur J Pharm Biopharm. 2014;88(1):186–93. 24747809 10.1016/j.ejpb.2014.04.002

[86] Li Z, Zhang Y, Zhu C, Guo T, Xia Q, Hou X, et al. Folic acid modified lipid-bilayer coated mesoporous silica nanoparticles co-loading paclitaxel and tanshinone IIA for the treatment of acute promyelocytic leukemia. Int J Pharm. 2020;586:119576. 32603839 10.1016/j.ijpharm.2020.119576

[87] Jiang X, Bugno J, Hu C, Yang Y, Herold T, Qi J, et al. Eradication of acute myeloid leukemia with FLT3 ligand-targeted miR-150 nanoparticles. Cancer Res. 2016;76(15):4470–80. 27280396 10.1158/0008-5472.CAN-15-2949 PMC4970973

[88] Park M, Vaikari VP, Lam AT, Zhang Y, MacKay JA, Alachkar H. Anti-FLT3 nanoparticles for acute myeloid leukemia: preclinical pharmacology and pharmacokinetics. J Control Release. 2020;324:317–29. 32428520 10.1016/j.jconrel.2020.05.021 PMC7473778

[89] Ali A, Phan A, Vaikari V, Park M, Pospiech M, Chu R, et al. FLT3/CD99 Bispecific antibody-based nanoparticles (BiAbs) for acute myeloid leukemia. Cancer Res Commun. 2024. 10.1158/2767-9764.CRC-24-0096 PMC11305399 39007347

[90] Abrams T, Connor A, Fanton C, Cohen SB, Huber T, Miller K, et al. Preclinical antitumor activity of a novel anti-c-KIT antibody-drug conjugate against mutant and wild-type c-KIT-Positive solid tumors. Clin Cancer Res. 2018;24(17):4297–308. 29764854 10.1158/1078-0432.CCR-17-3795

[91] Kim JO, Kim KH, Baek EJ, Park B, So MK, Ko BJ, et al. A novel anti-c-kit antibody-drug conjugate to treat wild-type and activating-mutant c-kit-positive tumors. Mol Oncol. 2022;16(6):1290–308. 34407310 10.1002/1878-0261.13084 PMC8936518

[92] Zhao N, Pei SN, Qi J, Zeng Z, Iyer SP, Lin P, et al. Oligonucleotide aptamer-drug conjugates for targeted therapy of acute myeloid leukemia. Biomaterials. 2015;67:42–51. 26204224 10.1016/j.biomaterials.2015.07.025 PMC4550516

[93] Zhang H, Luo J, Li Y, Henderson PT, Wang Y, Wachsmann-Hogiu S, et al. Characterization of high-affinity peptides and their feasibility for use in nanotherapeutics targeting leukemia stem cells. Nanomedicine. 2012;8(7):1116–24. 22197725 10.1016/j.nano.2011.12.004 PMC4577023

[94] Lin TY, Zhu Y, Li Y, Zhang H, Ma AH, Long Q, et al. Daunorubicin-containing CLL1-targeting nanomicelles have anti-leukemia stem cell activity in acute myeloid leukemia. Nanomedicine. 2019;20:102004. 31055076 10.1016/j.nano.2019.04.007 PMC8237247

[95] Carrion C, de Madariaga MA, Domingo JC. In vitro cytotoxic study of immunoliposomal doxorubicin targeted to human CD34(+) leukemic cells. Life Sci. 2004;75(3):313–28. 15135652 10.1016/j.lfs.2003.12.020

[96] Vaikari VP, Park M, Keossayan L, MacKay JA, Alachkar H. Anti-CD99 scFv-ELP nanoworms for the treatment of acute myeloid leukemia. Nanomedicine. 2020;29:102236. 32535112 10.1016/j.nano.2020.102236 PMC7508895

[97] Barth BM, IA E, Shanmugavelandy SS, Kaiser JM, Crespo-Gonzalez D, DiVittore NA, et al. Targeted indocyanine-green-loaded calcium phosphosilicate nanoparticles for in vivo photodynamic therapy of leukemia. ACS Nano. 2011;5(7):5325–37. 21675727 10.1021/nn2005766

[98] Kalinkovich A, Tavor S, Avigdor A, Kahn J, Brill A, Petit I, et al. Functional CXCR4-expressing microparticles and SDF-1 correlate with circulating acute myelogenous leukemia cells. Cancer Res. 2006;66(22):11013–20. 17108140 10.1158/0008-5472.CAN-06-2006

[99] Tavor S, Petit I, Porozov S, Avigdor A, Dar A, Leider-Trejo L, et al. CXCR4 regulates migration and development of human acute myelogenous leukemia stem cells in transplanted NOD/SCID mice. Cancer Res. 2004;64(8):2817–24. 15087398 10.1158/0008-5472.can-03-3693

[100] Zeng Z, Shi YX, Samudio IJ, Wang RY, Ling X, Frolova O, et al. Targeting the leukemia microenvironment by CXCR4 inhibition overcomes resistance to kinase inhibitors and chemotherapy in AML. Blood. 2009;113(24):6215–24. 18955566 10.1182/blood-2008-05-158311 PMC2699240

[101] Spoo AC, Lübbert M, Wierda WG, Burger JA. CXCR4 is a prognostic marker in acute myelogenous leukemia. Blood. 2007;109(2):786–91. 16888090 10.1182/blood-2006-05-024844

[102] Chen Y, Jacamo R, Konopleva M, Garzon R, Croce C, Andreeff M. CXCR4 downregulation of let-7a drives chemoresistance in acute myeloid leukemia. J Clin Invest. 2013;123(6):2395–407. 23676502 10.1172/JCI66553 PMC3668829

[103] Li L, Fang CJ, Ryan JC, Niemi EC, Lebrón JA, Björkman PJ, et al. Binding and uptake of H-ferritin are mediated by human transferrin receptor-1. Proc Natl Acad Sci USA. 2010;107(8):3505–10. 20133674 10.1073/pnas.0913192107 PMC2840523

[104] Gammella E, Buratti P, Cairo G, Recalcati S. The transferrin receptor: the cellular iron gate. Metallomics. 2017;9(10):1367–75. 28671201 10.1039/c7mt00143f

[105] Ponka P, Lok CN. The transferrin receptor: role in health and disease. Int J Biochem Cell Biol. 1999;31(10):1111–37. 10582342 10.1016/s1357-2725(99)00070-9

[106] Tortorella S, Karagiannis TC. Transferrin receptor-mediated endocytosis: a useful target for cancer therapy. J Membr Biol. 2014;247(4):291–307. 24573305 10.1007/s00232-014-9637-0

[107] Kawabata H. Transferrin and transferrin receptors update. Free Radic Biol Med. 2019;133:46–54. 29969719 10.1016/j.freeradbiomed.2018.06.037

[108] Liu Q, Wang M, Hu Y, Xing H, Chen X, Zhang Y, et al. Significance of CD71 expression by flow cytometry in diagnosis of acute leukemia. Leuk Lymphoma. 2014;55(4):892–98. 23962073 10.3109/10428194.2013.819100

[109] Wu B, Shi N, Sun L, Liu L. Clinical value of high expression level of CD71 in acute myeloid leukemia. Neoplasma. 2016;63(5):809–15. 27468886 10.4149/neo_2016_519

[110] Acharya S, Kala PS. Role of CD71 in acute leukemia- an immunophenotypic marker for erythroid lineage or proliferation? Indian J Pathol Microbiol. 2019;62(3):418–22. 31361230 10.4103/IJPM.IJPM_604_18

[111] Pande A, Dorwal P, Jain D, Tyagi N, Mehra S, Sachdev R, et al. Expression of CD71 by flow cytometry in acute leukemias: more often seen in acute myeloid leukemia. Indian J Pathol Microbiol. 2016;59(3):310–13. 27510666 10.4103/0377-4929.188145

[112] Neiveyans M, Melhem R, Arnoult C, Bourquard T, Jarlier M, Busson M, et al. A recycling anti-transferrin receptor-1 monoclonal antibody as an efficient therapy for erythroleukemia through target up-regulation and antibody-dependent cytotoxic effector functions. MAbs. 2019;11(3):593–605. 30604643 10.1080/19420862.2018.1564510 PMC6512944

[113] Kameda K, Yanagiya R, Miyatake Y, Carreras J, Higuchi H, Murayama H, et al. The hepatic niche leads to aggressive natural killer cell leukemia proliferation through the transferrin-transferrin receptor 1 axis. Blood. 2023;142(4):352–64. 37146246 10.1182/blood.2022018597

[114] Milatovich A, Kitamura T, Miyajima A, Francke U. Gene for the alpha-subunit of the human interleukin-3 receptor (IL3RA) localized to the X-Y pseudoautosomal region. Am J Hum Genet. 1993;53(5):1146–53. 8213838 PMC1682314

[115] Rapoport AP, DiPersio JF. Sequence analysis and functional studies of interleukin-3 receptor alpha subunit-encoding cDNAs amplified from KG-1 leukemic cells and normal human marrow. Gene. 1993;137(2):333–37. 8299967 10.1016/0378-1119(93)90030-7

[116] Blalock WL, Weinstein-Oppenheimer C, Chang F, Hoyle PE, Wang XY, Algate PA, et al. Signal transduction, cell cycle regulatory, and anti-apoptotic pathways regulated by IL-3 in hematopoietic cells: possible sites for intervention with anti-neoplastic drugs. Leukemia. 1999;13(8):1109–66. 10450743 10.1038/sj.leu.2401493

[117] Reddy EP, Korapati A, Chaturvedi P, Rane S. IL-3 signaling and the role of Src kinases, JAKs and STATs: a covert liaison unveiled. Oncogene. 2000;19(21):2532–47. 10851052 10.1038/sj.onc.1203594

[118] Muñoz L, Nomdedéu JF, López O, Carnicer MJ, Bellido M, Aventín A, et al. Interleukin-3 receptor alpha chain (CD123) is widely expressed in hematologic malignancies. Haematologica. 2001;86(12):1261–69. 11726317

[119] Testa U, Riccioni R, Militi S, Coccia E, Stellacci E, Samoggia P, et al. Elevated expression of IL-3Ralpha in acute myelogenous leukemia is associated with enhanced blast proliferation, increased cellularity, and poor prognosis. Blood. 2002;100(8):2980–88. 12351411 10.1182/blood-2002-03-0852

[120] Testa U, Pelosi E, Frankel A. CD 123 is a membrane biomarker and a therapeutic target in hematologic malignancies. Biomark Res. 2014;2(1):4. 24513123 10.1186/2050-7771-2-4 PMC3928610

[121] Bras AE, de Haas V, van Stigt A, Jongen-Lavrencic M, Beverloo HB, Te Marvelde JG, et al. CD123 expression levels in 846 acute leukemia patients based on standardized immunophenotyping. Cytometry B Clin Cytom. 2019;96(2):134–42. 30450744 10.1002/cyto.b.21745 PMC6587863

[122] Steelman LS, Algate PA, Blalock WL, Wang XY, Prevost KD, Hoyle PE, et al. Oncogenic effects of overexpression of the interleukin-3 receptor on hematopoietic cells. Leukemia. 1996;10(3):528–42. 8642872

[123] Du W, Li XE, Sipple J, Pang Q. Overexpression of IL-3Rα on CD34+CD38- stem cells defines leukemia-initiating cells in fanconi anemia AML. Blood. 2011;117(16):4243–52. 21330473 10.1182/blood-2010-09-309179 PMC3087476

[124] Vergez F, Green AS, Tamburini J, Sarry JE, Gaillard B, Cornillet-Lefebvre P, et al. High levels of CD34+CD38low/-CD123+ blasts are predictive of an adverse outcome in acute myeloid leukemia: a Groupe Ouest-Est des Leucemies Aigues et Maladies du Sang (GOELAMS) study. Haematologica. 2011;96(12):1792–98. 21933861 10.3324/haematol.2011.047894 PMC3232261

[125] Liu K, Zhu M, Huang Y, Wei S, Xie J, Xiao Y. CD123 and its potential clinical application in leukemias. Life Sci. 2015;122:59–64. 25445480 10.1016/j.lfs.2014.10.013

[126] Arai N, Homma M, Abe M, Baba Y, Murai S, Watanuki M, et al. Impact of CD123 expression, analyzed by immunohistochemistry, on clinical outcomes in patients with acute myeloid leukemia. Int J Hematol. 2019;109(5):539–44. 30847774 10.1007/s12185-019-02616-y

[127] El Achi H, Dupont E, Paul S, Khoury JD. CD123 as a biomarker in hematolymphoid malignancies: principles of detection and targeted therapies. Cancers (Basel). 2020;12(11). 10.3390/cancers12113087 PMC7690688 33113953

[128] O’Rourke K. CD123 expression linked to high-risk disease in children with acute myeloid leukemia. Cancer. 2022;128(7):1357–58. 35266674 10.1002/cncr.34152

[129] Hu J, Tang Z, Xu J, Ge W, Hu Q, He F, et al. The inhibitor of interleukin-3 receptor protects against sepsis in a rat model of cecal ligation and puncture. Mol Immunol. 2019;109:71–80. 30870654 10.1016/j.molimm.2019.03.002

[130] Zhao J, Wang M, Yang Y, Wang G, Che F, Li Q, et al. CD123 thioaptamer protects against sepsis via the blockade between IL-3/CD123 in a cecal ligation and puncture rat model. Nucleosides Nucleotides Nucleic Acids. 2021;40(1):16–31. 32985358 10.1080/15257770.2020.1815770

[131] Lesley J, Hyman R, Kincade PW. CD44 and its interaction with extracellular matrix. Adv Immunol. 1993;54:271–335. 8379464 10.1016/s0065-2776(08)60537-4

[132] Naor D, Sionov RV, Ish-Shalom D. CD44: structure, function, and association with the malignant process. Adv Cancer Res. 1997;71:241–319. 9111868 10.1016/s0065-230x(08)60101-3

[133] Khaldoyanidi S, Moll J, Karakhanova S, Herrlich P, Ponta H. Hyaluronate-enhanced hematopoiesis: two different receptors trigger the release of interleukin-1beta and interleukin-6 from bone marrow macrophages. Blood. 1999;94(3):940–49. 10419885

[134] Morimoto K, Robin E, Le Bousse-Kerdiles MC, Li Y, Clay D, Jasmin C, et al. CD44 mediates hyaluronan binding by human myeloid KG1A and KG1 cells. Blood. 1994;83(3):657–62. 7507730

[135] Smadja-Joffe F, Legras S, Girard N, Li Y, Delpech B, Bloget F, et al. CD44 and hyaluronan binding by human myeloid cells. Leuk Lymphoma. 1996;21(5–6):407–20, color plates following 528. 9172805 10.3109/10428199609093438

[136] Legras S, Levesque JP, Charrad R, Morimoto K, Le Bousse C, Clay D, et al. CD44-mediated adhesiveness of human hematopoietic progenitors to hyaluronan is modulated by cytokines. Blood. 1997;89(6):1905–14. 9058710

[137] Ayroldi E, Cannarile L, Migliorati G, Bartoli A, Nicoletti I, Riccardi C. CD44 (Pgp-1) inhibits CD3 and dexamethasone-induced apoptosis. Blood. 1995;86(7):2672–78. 7545465

[138] Naor D, Nedvetzki S, Golan I, Melnik L, Faitelson Y. CD44 in cancer. Crit Rev Clin Lab Sci. 2002;39(6):527–79. 12484499 10.1080/10408360290795574

[139] Bendall LJ, Bradstock KF, Gottlieb DJ. Expression of CD44 variant exons in acute myeloid leukemia is more common and more complex than that observed in normal blood, bone marrow or CD34+ cells. Leukemia. 2000;14(7):1239–46. 10914548 10.1038/sj.leu.2401830

[140] Allouche M, Charrad RS, Bettaieb A, Greenland C, Grignon C, Smadja-Joffe F. Ligation of the CD44 adhesion molecule inhibits drug-induced apoptosis in human myeloid leukemia cells. Blood. 2000;96(3):1187–90. 10910943

[141] Charrad RS, Gadhoum Z, Qi J, Glachant A, Allouche M, Jasmin C, et al. Effects of anti-CD44 monoclonal antibodies on differentiation and apoptosis of human myeloid leukemia cell lines. Blood. 2002;99(1):290–99. 11756184 10.1182/blood.v99.1.290

[142] Song G, Liao X, Zhou L, Wu L, Feng Y, Han ZC. HI44a, an anti-CD44 monoclonal antibody, induces differentiation and apoptosis of human acute myeloid leukemia cells. Leuk Res. 2004;28(10):1089–96. 15289023 10.1016/j.leukres.2004.02.005

[143] Orr SJ, Morgan NM, Elliott J, Burrows JF, Scott CJ, McVicar DW, et al. CD33 responses are blocked by SOCS3 through accelerated proteasomal-mediated turnover. Blood. 2007;109(3):1061–68. 17008544 10.1182/blood-2006-05-023556

[144] Ulyanova T, Blasioli J, Woodford-Thomas TA, Thomas ML. The sialoadhesin CD33 is a myeloid-specific inhibitory receptor. Eur J Immunol. 1999;29(11):3440–49. 10556798 10.1002/(SICI)1521-4141(199911)29:11<3440::AID-IMMU3440>3.0.CO;2-C

[145] Kraguljac N, Marisavljevic D, Jankovic G, Radosevic N, Pantic M, Donfrid M, et al. Characterization of CD13 and CD33 surface antigen-negative acute myeloid leukemia. Am J Clin Pathol. 2000;114(1):29–34. 10884796 10.1309/MFCP-7GMW-AQM4-ED3N

[146] Legrand O, Perrot JY, Baudard M, Cordier A, Lautier R, Simonin G, et al. The immunophenotype of 177 adults with acute myeloid leukemia: proposal of a prognostic score. Blood. 2000;96(3):870–77. 10910899

[147] Jilani I, Estey E, Huh Y, Joe Y, Manshouri T, Yared M, et al. Differences in CD33 intensity between various myeloid neoplasms. Am J Clin Pathol. 2002;118(4):560–66. 12375643 10.1309/1WMW-CMXX-4WN4-T55U

[148] De Propris MS, Raponi S, Diverio D, Milani ML, Meloni G, Falini B, et al. High CD33 expression levels in acute myeloid leukemia cells carrying the nucleophosmin (NPM1) mutation. Haematologica. 2011;96(10):1548–51. 21791474 10.3324/haematol.2011.043786 PMC3186318

[149] Clark MC, Stein A. CD33 directed bispecific antibodies in acute myeloid leukemia. Best Pract Res Clin Haematol. 2020;33(4):101224. 33279180 10.1016/j.beha.2020.101224

[150] Linenberger ML. CD33-directed therapy with gemtuzumab ozogamicin in acute myeloid leukemia: progress in understanding cytotoxicity and potential mechanisms of drug resistance. Leukemia. 2005;19(2):176–82. 15592433 10.1038/sj.leu.2403598

[151] Pollard JA, Alonzo TA, Loken M, Gerbing RB, Ho PA, Bernstein ID, et al. Correlation of CD33 expression level with disease characteristics and response to gemtuzumab ozogamicin containing chemotherapy in childhood AML. Blood. 2012;119(16):3705–11. 22378848 10.1182/blood-2011-12-398370 PMC3335378

[152] Liu J, Tong J, Yang H. Targeting CD33 for acute myeloid leukemia therapy. BMC Cancer. 2022;22(1):24. 34980040 10.1186/s12885-021-09116-5 PMC8722076

[153] Candoni A, Papayannidis C, Martinelli G, Simeone E, Gottardi M, Iacobucci I, et al. Flai (fludarabine, cytarabine, idarubicin) plus low-dose Gemtuzumab Ozogamicin as induction therapy in CD33-positive AML: final results and long term outcome of a phase II multicenter clinical trial. Am J Hematol. 2018;93(5):655–63. 29396857 10.1002/ajh.25057

[154] Yu B, Liu D. Gemtuzumab ozogamicin and novel antibody-drug conjugates in clinical trials for acute myeloid leukemia. Biomark Res. 2019;7:24. 31695916 10.1186/s40364-019-0175-x PMC6824118

[155] Schwemmlein M, Peipp M, Barbin K, Saul D, Stockmeyer B, Repp R, et al. A CD33-specific single-chain immunotoxin mediates potent apoptosis of cultured human myeloid leukaemia cells. Br J Haematol. 2006;133(2):141–51. 16611304 10.1111/j.1365-2141.2005.05869.x

[156] Mehta K, Shahid U, Malavasi F. Human CD38, a cell-surface protein with multiple functions. Faseb J. 1996;10(12):1408–17. 8903511 10.1096/fasebj.10.12.8903511

[157] Malavasi F, Deaglio S, Funaro A, Ferrero E, Horenstein AL, Ortolan E, et al. Evolution and function of the ADP ribosyl cyclase/CD38 gene family in physiology and pathology. Physiol Rev. 2008;88(3):841–86. 18626062 10.1152/physrev.00035.2007

[158] Chini EN. CD38 as a regulator of cellular NAD: a novel potential pharmacological target for metabolic conditions. Curr Pharm Des. 2009;15(1):57–63. 19149603 10.2174/138161209787185788 PMC2883294

[159] Hogan KA, Chini CCS, Chini EN. The multi-faceted ecto-enzyme CD38: roles in Immunomodulation, cancer, aging, and metabolic diseases. Front Immunol. 2019;10:1187. 31214171 10.3389/fimmu.2019.01187 PMC6555258

[160] Konopleva M, Rissling I, Andreeff M. CD38 in hematopoietic malignancies. Chem Immunol. 2000;75:189–206. 10851785 10.1159/000058769

[161] Lokhorst HM, Plesner T, Laubach JP, Nahi H, Gimsing P, Hansson M, et al. Targeting CD38 with daratumumab monotherapy in multiple myeloma. N Engl J Med. 2015;373(13):1207–19. 26308596 10.1056/NEJMoa1506348

[162] Sanchez L, Wang Y, Siegel DS, Wang ML. Daratumumab: a first-in-class CD38 monoclonal antibody for the treatment of multiple myeloma. J Hematol Oncol. 2016;9(1):51. 27363983 10.1186/s13045-016-0283-0 PMC4929758

[163] Drach J, Zhao S, Malavasi F, Mehta K. Rapid induction of CD38 antigen on myeloid leukemia cells by all trans-retinoic acid. Biochem Biophys Res Commun. 1993;195(2):545–50. 7690555 10.1006/bbrc.1993.2080

[164] Mehta K, McQueen T, Manshouri T, Andreeff M, Collins S, Albitar M. Involvement of retinoic acid receptor-alpha-mediated signaling pathway in induction of CD38 cell-surface antigen. Blood. 1997;89(10):3607–14. 9160665

[165] Sadasivan E, Rothenberg SP, da Costa M, Brink L. Characterization of multiple forms of folate-binding protein from human leukemia cells. Biochim Biophys Acta. 1986;882(3):311–21. 3460637 10.1016/0304-4165(86)90253-9

[166] Sadasivan E, da Costa M, Rothenberg SP, Brink L. Purification, properties, and immunological characterization of folate-binding proteins from human leukemia cells. Biochim Biophys Acta. 1987;925(1):36–47. 3474029 10.1016/0304-4165(87)90145-0

[167] Jaime-Ramirez AC, McMichael E, Kondadasula S, Skinner CC, Mundy-Bosse BL, Luedke E, et al. NK cell-mediated antitumor effects of a folate-conjugated immunoglobulin are enhanced by cytokines. Cancer Immunol Res. 2016;4(4):323–36. 26865456 10.1158/2326-6066.CIR-15-0168 PMC4818694

[168] Shen F, Ross JF, Wang X, Ratnam M. Identification of a novel folate receptor, a truncated receptor, and receptor type beta in hematopoietic cells: cDNA cloning, expression, immunoreactivity, and tissue specificity. Biochemistry. 1994;33(5):1209–15. 8110752 10.1021/bi00171a021

[169] Lu Y, Low PS. Folate-mediated delivery of macromolecular anticancer therapeutic agents. Adv Drug Deliv Rev. 2002;54(5):675–93. 12204598 10.1016/s0169-409x(02)00042-x

[170] Ross JF, Wang H, Behm FG, Mathew P, Wu M, Booth R, et al. Folate receptor type beta is a neutrophilic lineage marker and is differentially expressed in myeloid leukemia. Cancer. 1999;85(2):348–57. 10023702 10.1002/(sici)1097-0142(19990115)85:2<348::aid-cncr12>3.0.co;2-4

[171] Weinstein SJ, Hartman TJ, Stolzenberg-Solomon R, Pietinen P, Barrett MJ, Taylor PR, et al. Null association between prostate cancer and serum folate, vitamin B(6), vitamin B(12), and homocysteine. Cancer Epidemiol Biomarker Prev. 2003;12(11 Pt 1):1271–72. 14652294

[172] Kamen BA, Smith AK. A review of folate receptor alpha cycling and 5-methyltetrahydrofolate accumulation with an emphasis on cell models in vitro. Adv Drug Deliv Rev. 2004;56(8):1085–97. 15094208 10.1016/j.addr.2004.01.002

[173] Parker N, Turk MJ, Westrick E, Lewis JD, Low PS, Leamon CP. Folate receptor expression in carcinomas and normal tissues determined by a quantitative radioligand binding assay. Anal Biochem. 2005;338(2):284–93. 15745749 10.1016/j.ab.2004.12.026

[174] Low PS, Henne WA, Doorneweerd DD. Discovery and development of folic-acid-based receptor targeting for imaging and therapy of cancer and inflammatory diseases. Acc Chem Res. 2008;41(1):120–29. 17655275 10.1021/ar7000815

[175] Low PS, Kularatne SA. Folate-targeted therapeutic and imaging agents for cancer. Curr Opin Chem Biol. 2009;13(3):256–62. 19419901 10.1016/j.cbpa.2009.03.022

[176] Meshinchi S, Alonzo TA, Stirewalt DL, Zwaan M, Zimmerman M, Reinhardt D, et al. Clinical implications of FLT3 mutations in pediatric AML. Blood. 2006;108(12):3654–61. 16912228 10.1182/blood-2006-03-009233 PMC1895470

[177] Zorko NA, Bernot KM, Whitman SP, Siebenaler RF, Ahmed EH, Marcucci GG, et al. Mll partial tandem duplication and Flt3 internal tandem duplication in a double knock-in mouse recapitulates features of counterpart human acute myeloid leukemias. Blood. 2012;120(5):1130–36. 22674806 10.1182/blood-2012-03-415067 PMC3412333

[178] Takahashi S. Identification of Flt3 internal tandem duplications downstream targets by high-throughput immunoblotting protein array system. Am J Hematol. 2006;81(9):717–19. 16838337 10.1002/ajh.20697

[179] Lee SH, Paietta E, Racevskis J, Wiernik PH. Complete resolution of leukemia cutis with sorafenib in an acute myeloid leukemia patient with FLT3-ITD mutation. Am J Hematol. 2009;84(10):701–02. 19714594 10.1002/ajh.21511

[180] Tao S, Wang C, Chen Y, Deng Y, Song L, Shi Y, et al. Prognosis and outcome of patients with acute myeloid leukemia based on FLT3-ITD mutation with or without additional abnormal cytogenetics. Oncol Lett. 2019;18(6):6766–74. 31807186 10.3892/ol.2019.11051 PMC6876342

[181] Kiyoi H, Naoe T, Nakano Y, Yokota S, Minami S, Miyawaki S, et al. Prognostic implication of FLT3 and N-RAS gene mutations in acute myeloid leukemia. Blood. 1999;93(9):3074–80. 10216104

[182] Choi EJ, Lee JH, Lee JH, Park HS, Ko SH, Hur EH, et al. Comparison of anthracyclines used for induction chemotherapy in patients with FLT3-ITD-mutated acute myeloid leukemia. Leuk Res. 2018;68:51–56. 29544132 10.1016/j.leukres.2018.03.006

[183] Ozeki K, Kiyoi H, Hirose Y, Iwai M, Ninomiya M, Kodera Y, et al. Biologic and clinical significance of the FLT3 transcript level in acute myeloid leukemia. Blood. 2004;103(5):1901–08. 14604973 10.1182/blood-2003-06-1845

[184] Tsapogas P, Mooney CJ, Brown G, Rolink A. The cytokine Flt3-ligand in normal and malignant hematopoiesis. Int J Mol Sci. 2017;18(6). 10.3390/ijms18061115 PMC5485939 28538663

[185] Lennartsson J, Rönnstrand L. Stem cell factor receptor/c-Kit: from basic science to clinical implications. Physiol Rev. 2012;92(4):1619–49. 23073628 10.1152/physrev.00046.2011

[186] Pollard JA, Alonzo TA, Gerbing RB, Ho PA, Zeng R, Ravindranath Y, et al. Prevalence and prognostic significance of KIT mutations in pediatric patients with core binding factor AML enrolled on serial pediatric cooperative trials for de novo AML. Blood. 2010;115(12):2372–79. 20056794 10.1182/blood-2009-09-241075 PMC2845895

[187] Bühring HJ, Ullrich A, Schaudt K, Müller CA, Busch FW. The product of the proto-oncogene c-kit (P145c-kit) is a human bone marrow surface antigen of hemopoietic precursor cells which is expressed on a subset of acute non-lymphoblastic leukemic cells. Leukemia. 1991;5(10):854–60. 1720490

[188] Yamaguchi Y, Gunji Y, Nakamura M, Hayakawa K, Maeda M, Osawa H, et al. Expression of c-kit mRNA and protein during the differentiation of human hematopoietic progenitor cells. Exp Hematol. 1993;21(9):1233–38. 7687219

[189] Russkamp NF, Myburgh R, Kiefer JD, Neri D, Manz MG. Anti-CD117 immunotherapy to eliminate hematopoietic and leukemia stem cells. Exp Hematol. 2021;95:31–45. 33484750 10.1016/j.exphem.2021.01.003

[190] Broudy VC. Stem cell factor and hematopoiesis. Blood. 1997;90(4):1345–64. 9269751

[191] Yasuda A, Sawai H, Takahashi H, Ochi N, Matsuo Y, Funahashi H, et al. Stem cell factor/c-kit receptor signaling enhances the proliferation and invasion of colorectal cancer cells through the PI3K/Akt pathway. Dig Dis Sci. 2007;52(9):2292–300. 17410437 10.1007/s10620-007-9759-7

[192] Yasuda A, Sawai H, Takahashi H, Ochi N, Matsuo Y, Funahashi H, et al. The stem cell factor/c-kit receptor pathway enhances proliferation and invasion of pancreatic cancer cells. Mol Cancer. 2006;5:46. 17044945 10.1186/1476-4598-5-46 PMC1634869

[193] Ikeda H, Kanakura Y, Tamaki T, Kuriu A, Kitayama H, Ishikawa J, et al. Expression and functional role of the proto-oncogene c-kit in acute myeloblastic leukemia cells. Blood. 1991;78(11):2962–68. 1720040

[194] Wells SJ, Bray RA, Stempora LL, Farhi DC. CD117/CD34 expression in leukemic blasts. Am J Clin Pathol. 1996;106(2):192–95. 8712172 10.1093/ajcp/106.2.192

[195] Gao X, Lin J, Gao L, Deng A, Lu X, Li Y, et al. High expression of c-kit mRNA predicts unfavorable outcome in adult patients with t(8;21) acute myeloid leukemia. PLoS ONE. 2015;10(4):e0124241. 25860287 10.1371/journal.pone.0124241 PMC4393018

[196] Marshall AS, Willment JA, Lin HH, Williams DL, Gordon S, Brown GD. Identification and characterization of a novel human myeloid inhibitory C-type lectin-like receptor (MICL) that is predominantly expressed on granulocytes and monocytes. J Biol Chem. 2004;279(15):14792–802. 14739280 10.1074/jbc.M313127200

[197] Neumann K, Castiñeiras-Vilariño M, Höckendorf U, Hannesschläger N, Lemeer S, Kupka D, et al. Clec12a is an inhibitory receptor for uric acid crystals that regulates inflammation in response to cell death. Immunity. 2014;40(3):389–99. 24631154 10.1016/j.immuni.2013.12.015

[198] Chiffoleau E. C-Type lectin-like receptors as emerging orchestrators of sterile inflammation represent potential therapeutic targets. Front Immunol. 2018;9:227. 29497419 10.3389/fimmu.2018.00227 PMC5818397

[199] van Rhenen A, van Dongen GA, Kelder A, Rombouts EJ, Feller N, Moshaver B, et al. The novel AML stem cell associated antigen CLL-1 aids in discrimination between normal and leukemic stem cells. Blood. 2007;110(7):2659–66. 17609428 10.1182/blood-2007-03-083048

[200] Larsen H, Roug AS, Just T, Brown GD, Hokland P. Expression of the hMICL in acute myeloid leukemia-a highly reliable disease marker at diagnosis and during follow-up. Cytometry B Clin Cytom. 2012;82(1):3–8. 22173921 10.1002/cyto.b.20614

[201] Sutherland DR, Watt SM, Dowden G, Karhi K, Baker MA, Greaves MF, et al. Structural and partial amino acid sequence analysis of the human hemopoietic progenitor cell antigen CD34. Leukemia. 1988;2(12):793–803. 2462139

[202] Fina L, Molgaard HV, Robertson D, Bradley NJ, Monaghan P, Delia D, et al. Expression of the CD34 gene in vascular endothelial cells. Blood. 1990;75(12):2417–26. 1693532

[203] Silvestri F, Banavali S, Baccarani M, Preisler HD. The CD34 hemopoietic progenitor cell associated antigen: biology and clinical applications. Haematologica. 1992;77(3):265–73. 1385274

[204] Lapidot T, Sirard C, Vormoor J, Murdoch B, Hoang T, Caceres-Cortes J, et al. A cell initiating human acute myeloid leukaemia after transplantation into SCID mice. Nature. 1994;367(6464):645–48. 7509044 10.1038/367645a0

[205] Rombouts WJ, Martens AC, Ploemacher RE. Identification of variables determining the engraftment potential of human acute myeloid leukemia in the immunodeficient NOD/SCID human chimera model. Leukemia. 2000;14(5):889–97. 10803522 10.1038/sj.leu.2401777

[206] Costello R, Mallet F, Chambost H, Sainty D, Arnoulet C, Gastaut JA, et al. The immunophenotype of minimally differentiated acute myeloid leukemia (AML-M0): reduced immunogenicity and high frequency of CD34+/CD38- leukemic progenitors. Leukemia. 1999;13(10):1513–18. 10516751 10.1038/sj.leu.2401519

[207] Dick JE. Acute myeloid leukemia stem cells. Ann N Y Acad Sci. 2005;1044:1–5. 15958691 10.1196/annals.1349.001

[208] Kang LC, Dunphy CH. Immunoreactivity of MIC2 (CD99) and terminal deoxynucleotidyl transferase in bone marrow clot and core specimens of acute myeloid leukemias and myelodysplastic syndromes. Arch Pathol Lab Med. 2006;130(2):153–57. 16454553 10.5858/2006-130-153-IOMCAT

[209] Hahn JH, Kim MK, Choi EY, Kim SH, Sohn HW, Ham DI, et al. CD99 (MIC2) regulates the LFA-1/ICAM-1-mediated adhesion of lymphocytes, and its gene encodes both positive and negative regulators of cellular adhesion. J Immunol. 1997;159(5):2250–58. 9278313

[210] Chung SS, Eng WS, Hu W, Khalaj M, Garrett-Bakelman FE, Tavakkoli M, et al. CD99 is a therapeutic target on disease stem cells in myeloid malignancies. Sci Transl Med. 2017;9(374). 10.1126/scitranslmed.aaj2025 PMC5624309 28123069

[211] Manara MC, Pasello M, Scotlandi K. CD99: a cell surface protein with an oncojanus role in tumors. Genes (Basel). 2018;9(3). 10.3390/genes9030159 PMC5867880 29534016

[212] Travaglini S, Ottone T, Angelini DF, Fiori V, Dominici S, Noguera NI, et al. CD99 as a novel therapeutic target on leukemic progenitor cells in FLT3-ITD(mut) AML. Leukemia. 2022;36(6):1685–88. 35422094 10.1038/s41375-022-01566-5

[213] Hosen N, Park CY, Tatsumi N, Oji Y, Sugiyama H, Gramatzki M, et al. CD96 is a leukemic stem cell-specific marker in human acute myeloid leukemia. Proc Natl Acad Sci USA. 2007;104(26):11008–13. 17576927 10.1073/pnas.0704271104 PMC1904175

[214] Tavakkoli M, Chung SS, Park CY. Do preclinical studies suggest that CD99 is a potential therapeutic target in acute myeloid leukemia and the myelodysplastic syndromes? Expert Opin Ther Targets. 2018;22(5):381–83. 29637789 10.1080/14728222.2018.1464140

[215] Wang PL, O’Farrell S, Clayberger C, Krensky AM. Identification and molecular cloning of tactile. A novel human T cell activation antigen that is a member of the Ig gene superfamily. J Immunol. 1992;148(8):2600–08. 1313846

[216] Stanko K, Iwert C, Appelt C, Vogt K, Schumann J, Strunk FJ, et al. CD96 expression determines the inflammatory potential of IL-9-producing Th9 cells. Proc Natl Acad Sci USA. 2018;115(13):E2940–e9. 29531070 10.1073/pnas.1708329115 PMC5879650

[217] Li J, Xia Q, Di C, Li C, Si H, Zhou B, et al. Tumor cell-intrinsic CD96 mediates chemoresistance and cancer stemness by regulating mitochondrial fatty acid β-oxidation. Adv Sci (Weinh). 2023;10(7):e2202956. 36581470 10.1002/advs.202202956 PMC9982582

[218] Meads MB, Hazlehurst LA, Dalton WS. The bone marrow microenvironment as a tumor sanctuary and contributor to drug resistance. Clin Cancer Res. 2008;14(9):2519–26. 18451212 10.1158/1078-0432.CCR-07-2223

[219] Bernasconi P, Farina M, Boni M, Dambruoso I, Calvello C. Therapeutically targeting SELF-reinforcing leukemic niches in acute myeloid leukemia: a worthy endeavor? Am J Hematol. 2016;91(5):507–17. 26822317 10.1002/ajh.24312

[220] Ho TC, LaMere M, Stevens BM, Ashton JM, Myers JR, O’Dwyer KM, et al. Evolution of acute myelogenous leukemia stem cell properties after treatment and progression. Blood. 2016;128(13):1671–78. 27421961 10.1182/blood-2016-02-695312 PMC5043124

[221] Hu Q, Sun W, Wang J, Ruan H, Zhang X, Ye Y, et al. Conjugation of haematopoietic stem cells and platelets decorated with anti-PD-1 antibodies augments anti-leukaemia efficacy. Nat Biomed Eng. 2018;2(11):831–40. 31015615 10.1038/s41551-018-0310-2 PMC7032014

[222] Ci T, Li H, Chen G, Wang Z, Wang J, Abdou P, et al. Cryo-shocked cancer cells for targeted drug delivery and vaccination. Sci Adv. 2020;6(50). 10.1126/sciadv.abc3013 PMC7725453 33298439

[223] Li J, Wu H, Yu Z, Wang Q, Zeng X, Qian W, et al. Hematopoietic stem and progenitor cell membrane-coated vesicles for bone marrow-targeted leukaemia drug delivery. Nat Commun. 2024;15(1):5689. 38971796 10.1038/s41467-024-50021-9 PMC11227508

[224] Chen HT, Wang DY, Yao YY, Xiao YF, Zhao ZZ, Zhang ZQ, et al. Leukemia cell hitchhiking hypoxia responsive nanogel for improved immunotherapy of acute myeloid leukemia. Adv Funct Mater. 2024;34(48).

[225] Mann AP, Tanaka T, Somasunderam A, Liu X, Gorenstein DG, Ferrari M. E-selectin-targeted porous silicon particle for nanoparticle delivery to the bone marrow. Adv Mater. 2011;23(36):H278–82. 10.1002/adma.201101541 21833996

[226] Zong H, Sen S, Zhang G, Mu C, Albayati ZF, Gorenstein DG, et al. In vivo targeting of leukemia stem cells by directing parthenolide-loaded nanoparticles to the bone marrow niche. Leukemia. 2016;30(7):1582–86. 26669973 10.1038/leu.2015.343 PMC4911325

[227] Swart LE, Fens M, van Oort A, Waranecki P, Mata Casimiro LD, Tuk D, et al. Increased bone marrow uptake and accumulation of Very-late antigen-4 targeted lipid nanoparticles. Pharmaceutics. 2023;15(6). 10.3390/pharmaceutics15061603 PMC10304323 37376052

[228] Ho TC, Kim HS, Chen Y, Li Y, LaMere MW, Chen C, et al. Scaffold-mediated CRISPR-Cas9 delivery system for acute myeloid leukemia therapy. Sci Adv. 2021;7(21). 10.1126/sciadv.abg3217 PMC8133753 34138728

[229] Bae KH, Lai F, Mong J, Niibori-Nambu A, Chan KH, Her Z, et al. Bone marrow-targetable green tea catechin-based micellar nanocomplex for synergistic therapy of acute myeloid leukemia. J Nanobiotechnol. 2022;20(1):481. 10.1186/s12951-022-01683-4 PMC9670631 36384529

[230] Hao Wu YG, Ma J, Hu M, Xia J, Bao S, Liu Y, et al. Cytarabine delivered by CD44 and bone targeting redox-sensitive liposomes for treatment of acute myelogenous leukemia. Regenerative Biomater. 2022;9:11. 10.1093/rb/rbac058 PMC9469920 36110161

[231] Ackun-Farmmer MA, Soto CA, Lesch ML, Byun D, Yang L, Calvi LM, et al. Reduction of leukemic burden via bone-targeted nanoparticle delivery of an inhibitor of C-chemokine (C-C motif) ligand 3 (CCL3) signaling. Faseb J. 2021;35(4):e21402. 33724567 10.1096/fj.202000938RR PMC8594422

[232] Quesenberry PJ, Becker PS. Stem cell homing: rolling, crawling, and nesting. Proc Natl Acad Sci USA. 1998;95(26):15155–57. 9860935 10.1073/pnas.95.26.15155 PMC33927

[233] Vermeulen M, Le Pesteur F, Gagnerault MC, Mary JY, Sainteny F, Lepault F. Role of adhesion molecules in the homing and mobilization of murine hematopoietic stem and progenitor cells. Blood. 1998;92(3):894–900. 9680357

[234] Peled A, Kollet O, Ponomaryov T, Petit I, Franitza S, Grabovsky V, et al. The chemokine SDF-1 activates the integrins LFA-1, VLA-4, and VLA-5 on immature human CD34(+) cells: role in transendothelial/stromal migration and engraftment of NOD/SCID mice. Blood. 2000;95(11):3289–96. 10828007

[235] Lapidot T, Kollet O. The essential roles of the chemokine SDF-1 and its receptor CXCR4 in human stem cell homing and repopulation of transplanted immune-deficient NOD/SCID and NOD/SCID/B2m(null) mice. Leukemia. 2002;16(10):1992–2003. 12357350 10.1038/sj.leu.2402684

[236] Lapidot T, Dar A, Kollet O. How do stem cells find their way home? Blood. 2005;106(6):1901–10. 15890683 10.1182/blood-2005-04-1417

[237] Kollet O, Spiegel A, Peled A, Petit I, Byk T, Hershkoviz R, et al. Rapid and efficient homing of human CD34(+)CD38(-/low)CXCR4(+) stem and progenitor cells to the bone marrow and spleen of NOD/SCID and NOD/SCID/B2m(null) mice. Blood. 2001;97(10):3283–91. 11342460 10.1182/blood.v97.10.3283

[238] Jin L, Hope KJ, Zhai Q, Smadja-Joffe F, Dick JE. Targeting of CD44 eradicates human acute myeloid leukemic stem cells. Nat Med. 2006;12(10):1167–74. 16998484 10.1038/nm1483

[239] Chen FM, Wu LA, Zhang M, Zhang R, Sun HH. Homing of endogenous stem/progenitor cells for in situ tissue regeneration: promises, strategies, and translational perspectives. Biomaterials. 2011;32(12):3189–209. 21300401 10.1016/j.biomaterials.2010.12.032

[240] Chute JP. Stem cell homing. Curr Opin Hematol. 2006;13(6):399–406. 17053451 10.1097/01.moh.0000245698.62511.3d

[241] Lévesque JP, Helwani FM, Winkler IG. The endosteal ‘osteoblastic’ niche and its role in hematopoietic stem cell homing and mobilization. Leukemia. 2010;24(12):1979–92. 20861913 10.1038/leu.2010.214

[242] Khaldoyanidi S. Directing stem cell homing. Cell STEM Cell. 2008;2(3):198–200. 18371444 10.1016/j.stem.2008.02.012

[243] Weigel JA, Raymond RC, Weigel PH. The hyaluronan receptor for endocytosis (HARE) is not CD44 or CD54 (ICAM-1). Biochem Biophys Res Commun. 2002;294(4):918–22. 12061795 10.1016/S0006-291X(02)00558-2

[244] Harris EN, Weigel JA, Weigel PH. Endocytic function, glycosaminoglycan specificity, and antibody sensitivity of the recombinant human 190-kDa hyaluronan receptor for endocytosis (HARE). J Biol Chem. 2004;279(35):36201–09. 15208308 10.1074/jbc.M405322200

[245] Fraser JR, Appelgren LE, Laurent TC. Tissue uptake of circulating hyaluronic acid. A whole body autoradiographic study. Cell Tissue Res. 1983;233(2):285–93. 6413068 10.1007/BF00238296

[246] Courel MN, Maingonnat C, Bertrand P, Chauzy C, Smadja-Joffe F, Delpech B. Biodistribution of injected tritiated hyaluronic acid in mice: a comparison between macromolecules and hyaluronic acid-derived oligosaccharides. In Vivo. 2004;18(2):181–87. 15113045

[247] Ma S, Xu S, Li M, Du Y, Tian G, Deng J, et al. A bone targeting nanoparticle loaded OGP to restore bone homeostasis for osteoporosis therapy. Adv Healthc Mater. 2023;12(25):e2300560. 37562069 10.1002/adhm.202300560

[248] Sun X, Lin Y, Zhong X, Fan C, Liu Z, Chen X, et al. Alendronate-functionalized polymeric micelles target icaritin to bone for mitigating osteoporosis in a rat model. J Control Release. 2024;376:37–51. 39368708 10.1016/j.jconrel.2024.10.002

[249] Fu L, Zhang W, Zhou X, Fu J, He C. Tumor cell membrane-camouflaged responsive nanoparticles enable MRI-guided immuno-chemodynamic therapy of orthotopic osteosarcoma. Bioact Mater. 2022;17:221–33. 35386464 10.1016/j.bioactmat.2022.01.035 PMC8965157

[250] Sun K, Yuan L, Chen S, Sun Y, Wei D. Alendronate Pt(IV) prodrug amphiphile for enhanced chemotherapy targeting and bone destruction inhibition in osteosarcoma. Adv Healthc Mater. 2024;13(7):e2302746. 37988194 10.1002/adhm.202302746

[251] Abou-Elnour FS, El-Habashy SE, Essawy MM, Abdallah OY. Alendronate/lactoferrin-dual decorated lipid nanocarriers for bone-homing and active targeting of ivermectin and methyl dihydrojasmonate for leukemia. Biomater Adv. 2024;162:213924. 38875802 10.1016/j.bioadv.2024.213924

[252] Wu X, Hu Z, Nizzero S, Zhang G, Ramirez MR, Shi C, et al. Bone-targeting nanoparticle to co-deliver decitabine and arsenic trioxide for effective therapy of myelodysplastic syndrome with low systemic toxicity. J Control Release. 2017;268:92–101. 29042320 10.1016/j.jconrel.2017.10.012 PMC5722672

[253] Swami A, Reagan MR, Basto P, Mishima Y, Kamaly N, Glavey S, et al. Engineered nanomedicine for myeloma and bone microenvironment targeting. Proc Natl Acad Sci USA. 2014;111(28):10287–92. 24982170 10.1073/pnas.1401337111 PMC4104924

[254] Han TY, Hou LS, Li JX, Huan ML, Zhou SY, Zhang BL. Bone targeted miRNA delivery system for miR-34a with enhanced anti-tumor efficacy to bone-associated metastatic breast cancer. Int J Pharm. 2023;635:122755. 36801480 10.1016/j.ijpharm.2023.122755

[255] Gao H, Zhang J, Kleijn TG, Wu Z, Liu B, Ma Y, et al. Dual ligand-targeted Pluronic P123 polymeric micelles enhance the therapeutic effect of breast cancer with bone metastases. Oncol Res. 2024;32(4):769–84. 38560569 10.32604/or.2023.044276 PMC10972726

[256] Long M, Liu X, Huang X, Lu M, Wu X, Weng L, et al. Alendronate-functionalized hypoxia-responsive polymeric micelles for targeted therapy of bone metastatic prostate cancer. J Control Release. 2021;334:303–17. 33933517 10.1016/j.jconrel.2021.04.035

[257] Zhang X, Liu Q, Zhang T, Gao P, Wang H, Yao L, et al. Bone-targeted nanoplatform enables efficient modulation of bone tumor microenvironment for prostate cancer bone metastasis treatment. Drug Deliv. 2022;29(1):889–905. 35285760 10.1080/10717544.2022.2050845 PMC8928789

[258] Sheu TJ, Schwarz EM, O’Keefe RJ, Rosier RN, Puzas JE. Use of a phage display technique to identify potential osteoblast binding sites within osteoclast lacunae. J Bone Min Res. 2002;17(5):915–22. 10.1359/jbmr.2002.17.5.915 12009023

[259] Wang Y, Newman MR, Ackun-Farmmer M, Baranello MP, Sheu TJ, Puzas JE, et al. Fracture-targeted delivery of β-catenin agonists via peptide-functionalized nanoparticles augments Fracture Healing. ACS Nano. 2017;11(9):9445–58. 28881139 10.1021/acsnano.7b05103 PMC5736386

[260] Xiao B, Liu Y, Chandrasiri I, Adjei-Sowah E, Mereness J, Yan M, et al. Bone-targeted nanoparticle drug delivery system-mediated macrophage modulation for enhanced Fracture Healing. Small. 2024;20(7):e2305336. 37797180 10.1002/smll.202305336 PMC10922143

[261] Patra JK, Das G, Fraceto LF, Campos EVR, Rodriguez-Torres MDP, Acosta-Torres LS, et al. Nano based drug delivery systems: recent developments and future prospects. J Nanobiotechnol. 2018;16(1):71. 10.1186/s12951-018-0392-8 PMC6145203 30231877

[262] Torchilin VP. Multifunctional nanocarriers. Adv Drug Deliv Rev. 2006;58(14):1532–55. 17092599 10.1016/j.addr.2006.09.009

[263] Zhu S. CAR-T in cancer therapeutics and updates. J Natl Cancer Cent. 2024;4(3):189–94. 39281717 10.1016/j.jncc.2024.01.001 PMC11402450

[264] Frey NV. Chimeric antigen receptor T cells for acute lymphoblastic leukemia. Am J Hematol. 2019;94(S1):S24–S27. 30784101 10.1002/ajh.25442

[265] Ishikawa F, Yoshida S, Saito Y, Hijikata A, Kitamura H, Tanaka S, et al. Chemotherapy-resistant human AML stem cells home to and engraft within the bone-marrow endosteal region. Nat Biotechnol. 2007;25(11):1315–21. 17952057 10.1038/nbt1350

[266] Hanekamp D, Cloos J, Schuurhuis GJ. Leukemic stem cells: identification and clinical application. Int J Hematol. 2017;105(5):549–57. 28357569 10.1007/s12185-017-2221-5

[267] Villatoro A, Konieczny J, Cuminetti V, Arranz L. Leukemia stem cell release from the stem cell niche to treat acute myeloid leukemia. Front Cell Dev Biol. 2020;8:607. 32754595 10.3389/fcell.2020.00607 PMC7367216

[268] Lévesque JP, Winkler IG, Hendy J, Williams B, Helwani F, Barbier V, et al. Hematopoietic progenitor cell mobilization results in hypoxia with increased hypoxia-inducible transcription factor-1 alpha and vascular endothelial growth factor a in bone marrow. STEM Cells. 2007;25(8):1954–65. 17478585 10.1634/stemcells.2006-0688

[269] Zhang J, Ye J, Ma D, Liu N, Wu H, Yu S, et al. Cross-talk between leukemic and endothelial cells promotes angiogenesis by VEGF activation of the Notch/Dll4 pathway. Carcinogenesis. 2013;34(3):667–77. 23239744 10.1093/carcin/bgs386

